# Targeting Caveolin‐1 in Multiple Myeloma Cells Enhances Chemotherapy and Natural Killer Cell‐Mediated Immunotherapy

**DOI:** 10.1002/advs.202408373

**Published:** 2024-12-04

**Authors:** Dewen Zhan, Zhimin Du, Shang Zhang, Juanru Huang, Jian Zhang, Hui Zhang, Zhongrui Liu, Eline Menu, Jinheng Wang

**Affiliations:** ^1^ The Affiliated Traditional Chinese Medicine Hospital Guangzhou Medical University Guangzhou 510130 China; ^2^ Guangzhou Municipal and Guangdong Provincial Key Laboratory of Protein Modification and Degradation School of Basic Medical Sciences Guangzhou Medical University Guangzhou 511436 China; ^3^ School of Nursing Guangzhou Medical University Guangzhou 510182 China; ^4^ School of Biomedical Engineering Guangzhou Medical University Guangzhou 511436 China; ^5^ Department of Hematology and Immunology Myeloma Center Brussels Vrije Universiteit Brussel Brussels B‐1090 Belgium

**Keywords:** caveolin‐1, glutathione metabolism, immunotherapy, multiple myeloma, natural killer cell, redox homeostasis

## Abstract

The cell membrane transport capacity and surface targets of multiple myeloma (MM) cells heavily influence chemotherapy and immunotherapy. Here, it is found that caveolin‐1 (CAV1), a primary component of membrane lipid rafts and caveolae, is highly expressed in MM cells and is associated with MM progression and drug resistance. CAV1 knockdown decreases MM cell adhesion to stromal cells and attenuates cell adhesion‐mediated drug resistance to bortezomib. CAV1 inhibition in MM cells enhances natural killer cell‐mediated cytotoxicity through increasing CXCL10, SLAMF7, and CD112. CAV1 suppression reduces mitochondrial membrane potential, increases reactive oxygen species, and inhibits autophagosome‐lysosome fusion, resulting in the disruption of redox homeostasis. Additionally, CAV1 knockdown enhances glutamine addiction by increasing ASCT2 and LAT1 and dysregulates glutathione metabolism. As a result of CAV1 inhibition, MM cells are more sensitive to starvation, glutamine depletion, and glutamine transporter inhibition, and grow more slowly in vivo in a mouse model treated with bortezomib. The observation that CAV1 inhibition modulated by 6‐mercaptopurine, daidzin, and statins enhances the efficacy of bortezomib in vitro and in vivo highlights the translational significance of these FDA‐approved drugs in improving MM outcomes. These data demonstrate that CAV1 serves as a potent therapeutic target for enhancing chemotherapy and immunotherapy for MM.

## Graphical abstract

Caveolin‐1 (CAV1) is highly expressed in multiple myeloma (MM) cells and is associated with drug resistance. CAV1 knockdown reduces cell adhesion, enhances natural killer (NK) cell cytotoxicity, disrupts redox balance, and increases glutamine dependency. Inhibiting CAV1 improves the efficacy of bortezomib and NK cell‐based immunotherapy, underscoring its potential as a therapeutic target to enhance MM treatment.

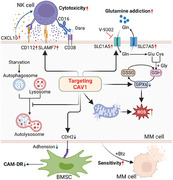

## 1 Introduction

In clinical practice or clinical trials, proteasome inhibitors, immunomodulatory drugs, and monoclonal antibodies (MoAbs) against CD38 or SLAMF7 have been combined to achieve significant remission rates in newly diagnosed and relapsed multiple myeloma (MM) patients.^[^ ^1^ ^]^ Unfortunately, however, in most MM patients, relapse is unavoidable due to cell‐intrinsic heterogeneity, clonal evolution, and/or acquired resistance.^[^ ^2^ ^]^ Therefore, novel biological targeted therapies are still urgently needed to improve MM treatment.

As well as triggering apoptosis, endocytosis, cholesterol transport, signal transduction, and immune responses, membrane lipid rafts also influence the efficacy of chemotherapy and immunotherapy by controlling cell membrane transport capacity and surface targets.^[^ ^3^ ^]^ The integral membrane protein caveolin‐1 (CAV‐1) is a primary component of lipid rafts and mediates cellular processes dependent on lipid raft structures.^[^ ^4^ ^]^ As a major structural protein in cholesterol‐rich caveolae, CAV1 modulates cellular signal transduction by regulating endocytosis and recycling of membrane proteins.^[^ ^4^, ^5^ ^]^ It has been shown that CAV1 promotes the endocytosis of HER‐2, an immunotherapy target for breast cancer, and thus attenuates the binding and therapeutic efficacy of anti‐HER2 MoAb trastuzumab.^[^ ^6^ ^]^ Many cancer types are associated with CAV‐1 expression, which plays an essential role in regulating cell growth, survival, endocytosis, and migration.^[^ ^7^ ^]^ CAV‐1 overexpression was found in both MM cell lines and patient myeloma cells and may contribute to MM cell growth and migration.^[^ ^8^ ^]^ However, its biological and clinical implications in MM are still not fully elucidated.

In this work, we comprehensively demonstrated the diverse roles of CAV1 in modulating MM cell adhesion, natural killer (NK) cell‐mediated cytotoxicity, glutaminolysis, and reactive oxygen species (ROS) homeostasis and validated CAV1 as a potent target for enhancing the efficacy of bortezomib‐ and NK cell‐based anti‐MM therapy.

## 2 Results

### 2.1 CAV1 Knockdown Reduces MM Cell Adhesion and Adhesion‐Mediated Drug Resistance (CAM‐DR)

**Figure 1:**
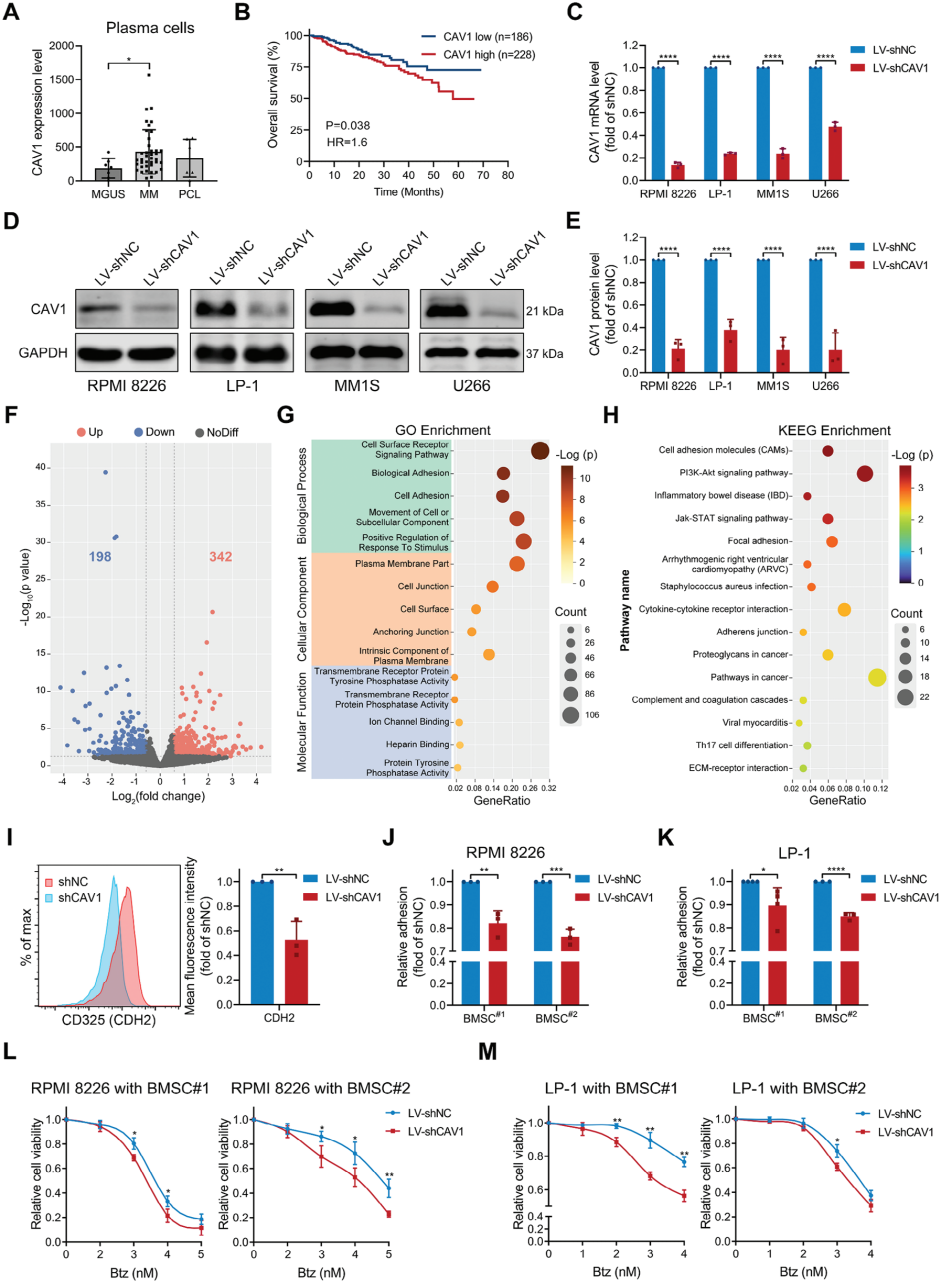
CAV1 knockdown attenuates cell adhesion to BMSCs and adhesion‐mediated drug resistance to bortezomib. A) CAV1 expression (GSE2113) in CD138^+^ plasma cells from patients with monoclonal gammopathy of undetermined significance (MGUS, n = 7), multiple myeloma (MM, n = 39), and plasma cell leukemia (PCL, n = 6). The vertical axis represents the normalized signal expression value. B) Overall survival according to high and low CAV1 expression in plasma cells was determined in MM patients (GSE4581) using the Log‐rank (Mantel‐Cox) test (optimal cut‐off points). C) mRNA expression of CAV1 in MM cells transduced with lentivirus expressing shRNAs against NC (LV‐shNC) or CAV1 (LV‐shCAV1) was determined using qRT‐PCR. D and E) Protein expression of CAV1 in MM cells expressing shNC or shCAV1 was detected using a western blot. GAPDH was included as a loading control. F) Volcano plot showing the differential mRNA expression between RPMI 8226 expressing shNC and shCAV1. Red dots represent up‐regulated genes and blue dots represent down‐regulated genes. G) Bubble plots showing the top five GO functional enrichment of genes significantly regulated by CAV1 in RPMI 8226 cells. H) Bubble plots showing the top 15 KEGG pathways of genes significantly regulated by CAV1 in RPMI 8226 cells. I) The surface level of CD325 (CDH2) in MM cells expressing shNC or shCAV1 was determined using flow cytometry. J and K) Adhesion assays of (J) RPMI 8226 or (K) LP‐1 cells expressing shNC or shCAV1 in co‐culture with BMSCs derived from MM patients. L) RPMI 8226 or (M) LP‐1 cells expressing shNC or shCAV1 were cultured with BMSCs derived from MM patients in the presence of bortezomib (Btz) at the indicated concentration for 48 h and the cell viability was measured. *p*‐values were calculated with an unpaired two‐sided Student *t*‐test. Error bar, mean ± SD. ^*^, *p* < 0.05; ^**^, *p* < 0.01; ^***^, *p* < 0.001; ^****^, *p* < 0.0001.

First, CAV1 expression was evaluated in plasma cells obtained from healthy donors (HD) and patients with monoclonal gammopathy of undetermined significance (MGUS) or MM. According to publicly available microarray datasets, the levels of CAV1 were significantly higher in CD138+ plasma cells obtained from MM patients compared to those from HD or patients with MGUS (**Figure** **1**A; Figure S1A, Supporting Information). High expression of CAV1 was significantly correlated with the shorter overall survival (OS) and progression‐free survival (PFS) of MM patients, especially those patients treated with bortezomib (Figure 1B; Figure S1B,C, Supporting Information). To determine the role of CAV1 in MM progression, four MM cell lines, including RPMI 8226, MM1S, LP‐1, and U266, were transduced with lentiviruses expressing shRNAs targeting CAV1. The mRNA and protein expression of CAV1 were significantly knocked down by shRNAs (Figure 1C–E). However, CAV1 knockdown did not affect the growth and cell cycle of MM cells in vitro (Figure S1D–F, Supporting Information). Inhibition of CAV1 enhanced the chemosensitivity to bortezomib in LP‐1 cells, but not in RPMI 8226 cells (Figure S1G,H, Supporting Information). RNA sequencing was next used to investigate the function of CAV1 in MM cells. Knockdown of CAV1 significantly induced up‐regulation of 342 genes and down‐regulation of 198 genes in RPMI 8226 cells (Figure 1F). CAV1 inhibition mainly regulated genes involved in the cell surface receptor pathway, biological adhesion, and cell adhesion (Figure 1G), and was associated with cell adhesion molecules, PI3K‐Akt and Jak‐STAT signaling pathways, and focal adhesion (Figure 1H). Cell surface, receptor complex, cell adhesion mediator activity, and adherens junction were significantly enriched in and positively correlated with CAV1 inhibition (Figure S2A, Supporting Information), suggesting a close relationship between CAV1 and cell adhesion. Moreover, GSVA analysis revealed that a variety of cell processes or pathways were dysregulated by CAV1 knockdown (Figure S2B,C, Supporting Information). As one of the adhesion‐related genes regulated by CAV1 (Figure S2D, Supporting Information), CDH2 has been reported to contribute to the adhesion of MM cells to bone marrow stromal cells (BMSCs).^[^ ^9^ ^]^ CAV1 knockdown significantly reduced the mRNA and surface expression of CDH2 (CD325) in MM cells (Figure 1I; Figure S2E, Supporting Information). Low expression of CDH2 was significantly correlated with longer OS of MM patients (Figure S2F, Supporting Information). As a result, CAV1 inhibition decreased the adhesion of MM cells to BMSCs (Figure 1J,K) and enhanced chemosensitivity to bortezomib in MM cells co‐cultured with BMSCs (Figure 1L,M). These results indicate the involvement of CAV1 in MM cell adhesion and regulation of CAM‐DR.

### 2.2 CAV1 Inhibition in MM Cells Enhances NK Cell‐Mediated Cytotoxicity

**Figure 2:**
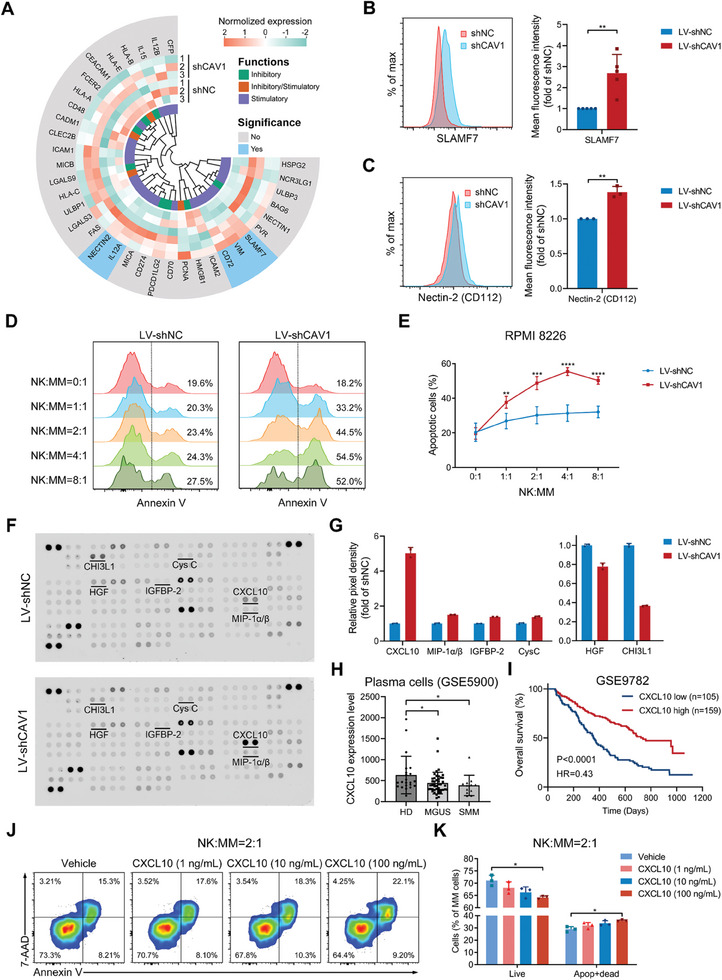
CAV1 regulates the expression of SLAMF7, Nectin‐2, and CXCL10, and modulates NK cell‐mediated cytotoxicity. A) A circular heatmap representing the mRNA expression of NK‐related inhibitory or stimulatory molecules in MM cells expressing shNC or shCAV1. B and C) The surface level of (B) SLAMF7 and (C) Nectin‐2 (CD112) in MM cells expressing shNC or shCAV1 was determined using flow cytometry. D and E) NK‐92 cells were co‐cultured with MM cells expressing shNC or shCAV1 at the indicated ratio for 12 h, and the apoptotic (Annexin V^+^) cells were determined using flow cytometry. F and G) Human cytokine array of conditioned medium obtained from RPMI 8226 cells expressing shNC or shCAV1. H) CXCL10 expression (GSE5900) in CD138^+^ plasma cells from HD (n = 22), MGUS (n = 44), and smoldering MM (SMM, *n* = 12). I) Overall survival according to high and low CXCL10 expression on MM cells was determined in MM patients (GSE9782). J and K) NK92 cells were co‐cultured with RPMI 8226 cells in the presence of CXCL10 at the indicated concentrations for 12 h. The apoptotic (Annexin V^+^) cells were determined using flow cytometry. *p*‐values were calculated with unpaired two‐sided Student *t*‐test or one‐way ANOVA test. Apop, apoptotic. Error bar, mean ± SD. ^*^, *p* < 0.05; ^**^, *p* < 0.01; ^***^, *p* < 0.001; ^****^, *p* < 0.0001.

CAV1 is closely associated with cell adhesion and its inhibition may regulate NK cell‐mediated MM cell killing through modulating surface ligands. Thus, genes involved in NK cell activation or inhibition in CAV1‐deficient MM cells were analyzed after RNA sequencing (**Figure** **2**A; Figure S3A, Supporting Information). Among them, the mRNA and surface expression of SLAMF7 was significantly increased by CAV1 knockdown (Figure 2B; Figure S3B, Supporting Information). High expression of SLAMF7 was significantly correlated with longer OS and PFS of MM patients, especially bortezomib‐treated patients (Figure S3C,D, Supporting Information). In addition, the surface level of Nectin‐2 (CD112) was also elevated by CAV1 depletion (Figure 2C). Nectin‐2 was reduced in CD138^+^ plasma cells obtained from MM patients compared to those from HD or MGUS patients and was positively correlated with the OS of MM patients treated with bortezomib in Mulligan's cohort (Figure S3E,F, Supporting Information). Additionally, CAV1 knockdown increased the cell surface death receptor Fas, which was also positively correlated with the PFS of bortezomib‐treated MM patients in Mulligan's cohort (Figure S3G,H, Supporting Information). In addition, surface CD38 was increased by CAV1 knockdown in these MM cells (Figure S3I, Supporting Information). To determine whether increased levels of these surface molecules by CAV1 knockdown benefit NK cell‐mediated cell cytotoxicity, MM cells were co‐cultured with NK‐92 cells. CAV1 inhibition in MM cells significantly enhanced NK cell‐mediated MM cell killing (Figure 2D,E).

Moreover, cytokines regulated by CAV1 were examined using a cytokine array (Figure 2F). CAV1 knockdown upregulated the release of CXCL10, MIP‐1α/β, IGFBP‐2, and cystatin C, and reduced the secretion of HGF and CHI3L1 (Figure 2G). Among them, CXCL10 was increased fivefold by CAV1 inhibition. CXCL10 was decreased in CD138^+^ plasma cells obtained from MM patients compared to those from HD or MGUS patients (Figure 2H; Figure S3J, Supporting Information), and was positively correlated with the OS and PFS of MM patients, especially bortezomib‐treated patients (Figure 2I; Figure S3K, Supporting Information). Furthermore, CXCL10 increased NK cell‐mediated MM cell killing in vitro (Figure 2J,K). These data suggest that targeting CAV1 in MM cells enhances NK cell‐mediated cytotoxicity against MM cells, which may be facilitated by increased SLAMF7, CD112, and CXCL10.

### 2.3 Targeting CAV1 Enhances Primary NK Cell‐Mediated Cytotoxicity and Daratumumab Efficacy

**Figure 3:**
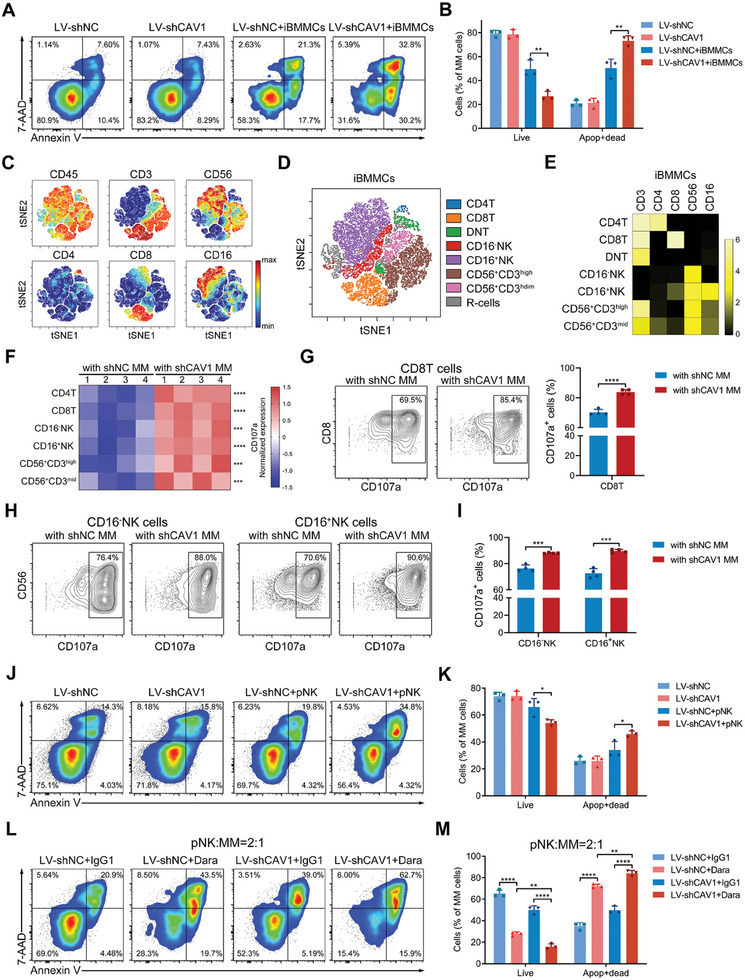
Targeting CAV1 enhances NK cell‐mediated cytotoxicity and improves daratumumab efficiency. A and B) In vitro BMMCs (iBMMCs, CD45^+^) were co‐culture with RPMI 8226 (CD45^−^) cells expressing shNC and shCAV1 at the ratio of 4:1 for six hours and the apoptosis of MM cells was determined using flow cytometry. C) viSNE maps colored by the normalized expression of the indicated markers in iBMMCs. D) viSNE map colored by eight cell populations after clustering. R‐cells, rest of cells. E) A heatmap showing the normalized median expression of five indicated markers in seven identified cell subsets. F) A heatmap showing the normalized mean expression of the CD107a in seven cell subsets. G) Representative contour plots for CD107a in CD8 T cells after co‐cultured with MM cells expressing shNC or shCAV1. H) Representative contour plots for CD107a in CD16^+^ or CD16^−^ NK cells after co‐cultured with MM cells expressing shNC or shCAV1. I) Bar plots showing the frequencies of the CD107a^+^ cells in NK cell subsets. J and K) Purified NK (pNK) cells were co‐culture with RPMI 8226 cells expressing shNC and shCAV1 at the ratio of 2:1 for six hours and the apoptosis of MM cells was determined using flow cytometry. L and M) In the presence of 10 µg mL^−1^ daratumumab (Dara) or isotype IgG1, pNK cells were co‐culture with RPMI 8226 cells expressing shNC and shCAV1 at the ratio of 2:1 for six hours and the apoptosis of MM cells were determined using flow cytometry. *p*‐values were calculated with unpaired two‐sided Student *t*‐test or one‐way ANOVA test. Apop, apoptotic. Error bar, mean ± SD. ^*^, *p* < 0.05; ^**^, *p* < 0.01; ^***^, *p* < 0.001; ^****^, *p* < 0.0001.

Next, we determined whether targeting CAV1 enhances the cytotoxicity of NK cells derived from MM patients. Bone marrow mononuclear cells (BMMC) were cultured in vitro (iBMMCs) with a serum‐free medium specific for NK cell expansion. After seven days of in vitro culture, the iBMMCs were mainly composed of NK cells and T cells. CAV1‐silenced MM cells exhibited increased cell death when co‐cultured with iBMMCs (**Figure** **3**A,B). Mass cytometry, a high‐throughput single‐cell technique, was then used to comprehensively examine the effector cells in these iBMMCs. viSNE analysis was used to visualize these high‐dimensional mass cytometry data in 2D,^[^ ^10^ ^]^ and seven T or NK cell subsets were identified (Figure 3C–E; Figure S4A, Supporting Information). CD107a, a marker for degranulation, was highly expressed in all identified cell subsets after co‐culturing with CAV1‐deficient MM cells (Figure 3F). The proportion of CD107a^+^ cells in CD8 T, CD4 T, CD16^+^NK, and CD16^−^NK cells was increased in the presence of CAV1‐depleted MM cells (Figure 3G–I; Figure S4B, Supporting Information). In addition, CD25, the receptor of an important stimulatory cytokine IL‐2, was also increased in CD8 T, CD16^+^NK, and CD16^−^NK cells (Figure S4C–F, Supporting Information). The mean expression of CD69, an early activation marker, was increased mainly in NK cells and CD56^+^CD3^high/dim^ cells, whereas the proportion of CD69^+^ cells in these cell subsets was not altered (Figure S4G,H, Supporting Information). Since iBMMCs contain NK cells expressing CD16 (Fcγ receptor III), they were co‐cultured with MM cells in the presence of daratumumab, a MoAb targeting CD38. CAV1 inhibition in MM cells also further enhanced iBMMC‐mediated cytotoxicity in the presence of daratumumab (Figure S4I,J, Supporting Information). NK cells were then purified from iBMMCs and cultured with MM cells. CAV1 knockdown enhanced purified NK cell‐mediated MM cell killing in the absence and presence of daratumumab (Figure 3J–M). Additionally, CAV1 knockdown increased NK cell‐mediated MM cell killing in the presence of elotuzumab, a MoAb targeting SLAMF7 (Figure S4K,L, Supporting Information). All these findings indicate that targeting CAV1 in MM cells boosts NK cell‐mediated cytotoxicity and antibody‐dependent cellular cytotoxicity.

### 2.4 CAV1 Suppression Inhibits MM in Vivo Growth and Regulates Redox Homeostasis and Autophagy

**Figure 4:**
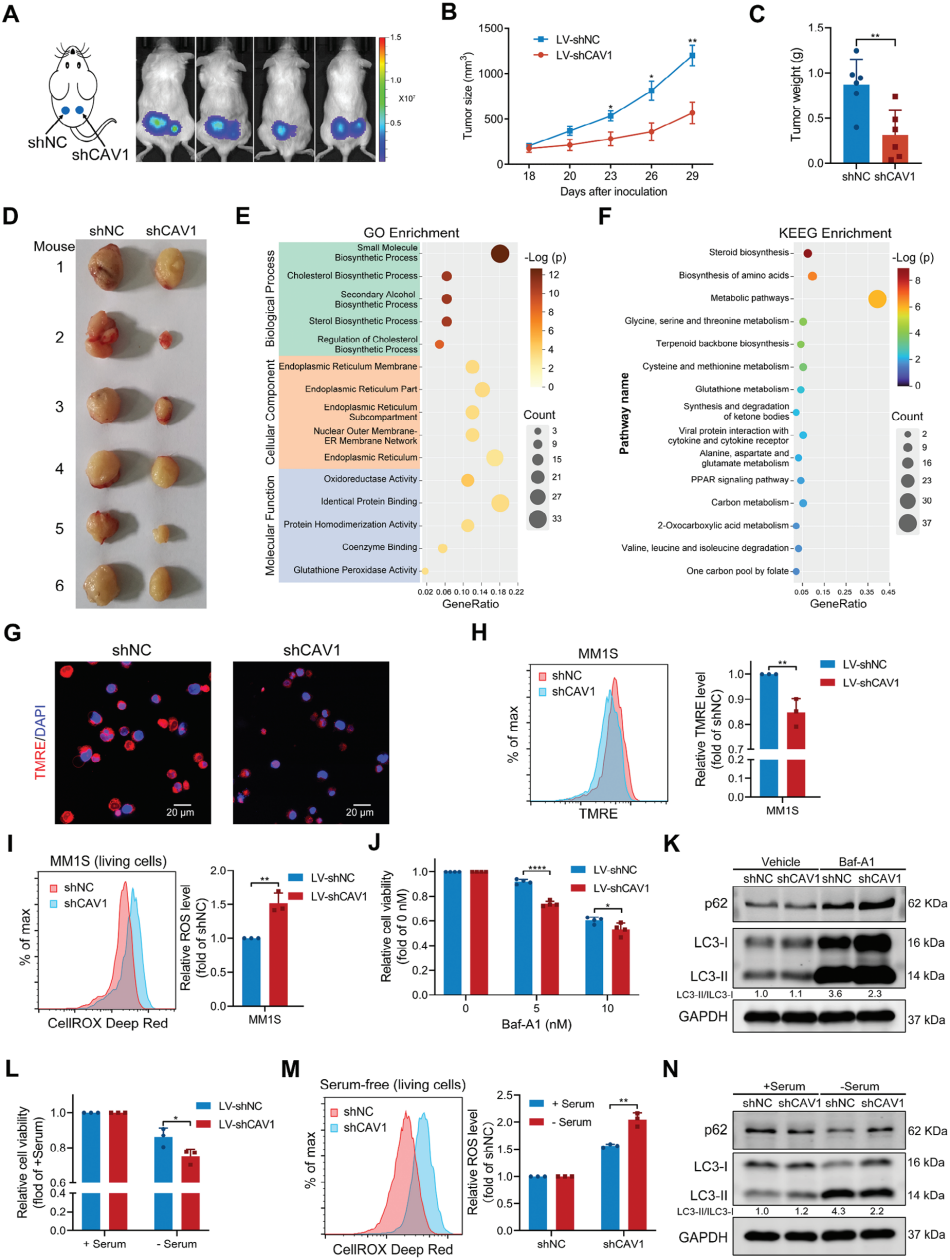
CAV1 knockdown inhibits MM cell growth in vivo and modulates ROS and the autophagy process. A) B‐NDG mice (n = 4) were subcutaneously inoculated with MM1S‐GFP‐Luc cells expressing shNC or shCAV1 in the left or right flank, respectively. Representative bioluminescence images of mice inoculated with MM cells. B–D) B‐NDG mice (n = 6) were subcutaneously inoculated with MM1S cells expressing shNC or shCAV1 in the left or right flank, respectively. (B) Tumor size of MM1S cells expressing shNC or shCAV1 at the indicated days were recorded. Error bar, mean ± SEM. (C and D) Tumor weight and images were obtained from B‐NDG mice subcutaneously inoculated with MM1S cells expressing shNC or shCAV1 for 31 days. E) Bubble plots showing the top five GO functional enrichment of genes significantly regulated by CAV1 in MM1S cells. F) Bubble plots showing the top 15 KEGG pathways of genes significantly regulated by CAV1 in MM1S cells. G) Representative confocal microscope images showing the levels of TMRE (red) in MM1S cells expressing shNC or shCAV1. DAPI (blue) was used to stain the cell nuclei. H) TMRE level in MM1S cells expressing shNC or shCAV1 was measured using flow cytometry. I) MM1S cells expressing shNC or shCAV1 were incubated with CellROX Deep Red for 15 min and the ROS level was determined using flow cytometry. J) MM1S cells expressing shNC or shCAV1 were treated with bafilomycin A1 (Baf‐A1) at the indicated concentrations for 48 h and the cell viability was measured. K) MM1S cells expressing shNC or shCAV1 were treated with Baf‐A1 (5 nM) for 48 h and the protein level of p62 and LC3‐I/II was detected using western blot. GAPDH was included as a loading control. L) MM1S cells expressing shNC or shCAV1 were cultured with or without serum for 24 h and cell viability was measured. M) MM1S cells expressing shNC or shCAV1 were cultured with or without serum for 24 h and incubated with CellROX Deep Red for 15 min. The ROS level was then determined using flow cytometry. N) MM1S cells expressing shNC or shCAV1 were cultured with or without serum for 24 h and the protein level of p62 and LC3‐1/II was detected using western blot. *p*‐values were calculated with unpaired two‐sided Student *t*‐test or Mann–Whitney U test. Error bar, mean ± SD. ^*^, *p* < 0.05; ^**^, *p* < 0.01; ^***^, *p* < 0.001; ^****^, *p* < 0.0001.

Although CAV1 knockdown did not affect MM growth in vitro, it significantly suppressed MM cell growth in an MM1S murine xenograft model (**Figure** **4**A–D). To explore the mechanisms involved in this in vivo inhibition, RNA sequencing was performed using MM1S cells. 151 genes and 102 genes were up‐regulated and down‐regulated, respectively, by CAV1 knockdown (Figure S5A, Supporting Information). The gene ontology (GO) analysis indicated that CAV1 is mainly related to small molecule biosynthetic process, endoplasmic reticulum, and oxidoreductase activity (Figure 4E). The Kyoto Encyclopedia of Genes and Genomes (KEGG) analysis revealed that it is mainly associated with steroid biosynthesis, biosynthesis of amino acids, and metabolic pathways (Figure 4F). The gene set enrichment analysis (GSEA) demonstrated that CAV1 is related to mitochondrion and ROS‐related processes (Figure S5B, Supporting Information) in MM1S cells. CAV1 inhibition decreased mitochondrial membrane potential and increased total and mitochondrial ROS levels (Figure 4G–I; Figure S5C,D, Supporting Information). GSEA analysis showed that CAV1 is also related to autophagy and cellular response to starvation (Figure S5E, Supporting Information). Thus, bafilomycin A1 (Baf‐A1), an inhibitor of autophagosome‐lysosome fusion, was used to examine whether CAV1 knockdown affects the autophagy process. CAV1 inhibition enhanced the sensitivity to Baf‐A1 in MM1S cells, reduced the LC3‐II/LC3‐I ratio, and increased the p62 level in the presence of Baf‐A1 (Figure 4J,K). CAV1 depletion also enhanced the sensitivity to starvation in MM1S cells and further increased the ROS in MM1S cells (Figure 4L,M). Moreover, the knockdown of CAV1 reduced the LC3‐II/LC3‐I ratio and increased the p62 level under starvation (Figure 4N). A reduced LC3‐II/LC3‐I ratio together with an increased p62 level suggests the inhibition of autophagosome‐lysosome fusion.^[^ ^11^ ^]^ Thus, CAV1 inhibition may impair cell viability through disturbing autophagy flux in the presence of Baf‐A1 and starvation. All these data indicate that targeting CAV1 regulates redox homeostasis and autophagy without inducing clear cell death in vitro, but inhibits MM cell growth in vivo.

### 2.5 CAV1 Depression Reprograms MM Cell Metabolism

**Figure 5:**
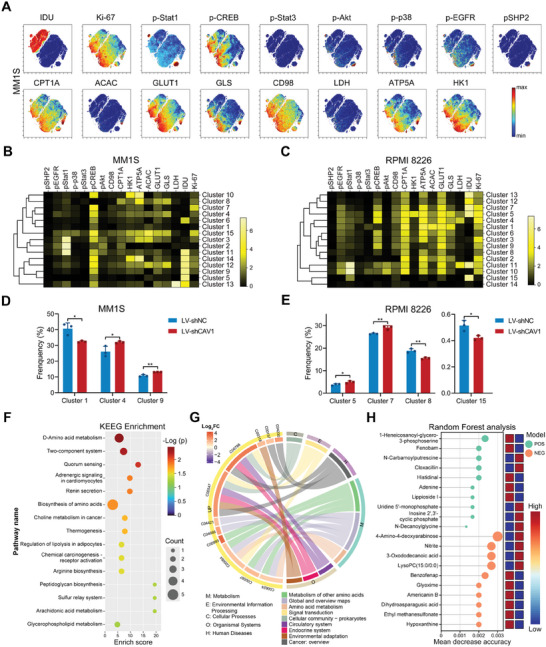
CAV1 inhibition reprograms the cell metabolism in MM cells. A–E) Multiple metabolism‐related proteins in MM1S cells expressing shNC or shCAV1 were analyzed using mass cytometry. (A) viSNE maps colored by the normalized expression of the indicated markers in MM1S cells. (B and C) A heatmap showing the normalized mean expression of the indicated markers in 15 FlowSOM clusters of (B) MM1S or (C) RPMI 8226 cells. (D and E) Bar plots showing the frequencies of the indicated clusters in (D) MM1S or (E) RPMI 8226 cells expressing shNC or shCAV1. F) Bubble plots showing the top 15 KEGG pathways of metabolites significantly regulated by CAV1 in MM1S cells. G) Chordal graph showing the relationships between KEGG pathways and metabolites significantly regulated by CAV1 in MM1S cells. H) Lollipop Chart showing the top 10 import metabolites under POS and NEG model for distinguishing MM1S cells expressing shNC and shCAV. *p*‐values were calculated with an unpaired two‐sided Student *t*‐test. Error bar, mean ± SD. ^*^, *p* < 0.05; ^**^, *p* < 0.01.

Based on our in vitro findings and sequencing data, we sought to investigate whether CAV1 inhibition reprograms the metabolism in MM. Mass cytometry was employed to measure the phosphorylation level of pathway proteins and key proteins that regulate glucose and energy metabolism at the single‐cell level. Using viSNE algorithm analysis, a heterogeneity of cells in MM cell lines was clearly observed (**Figure** **5**A; Figure S6A, Supporting Information). 15 clusters with distinct pathway activations and metabolic features in MM1S or RPMI 8226 cells were identified using FlowSOM (Figure S6B,C, Supporting Information), a powerful clustering algorithm that builds self‐organizing maps.^[^ ^12^ ^]^ These clusters have distinct features in the levels of phosphorylated proteins regulating key signaling pathways and key enzymes modulating cell metabolism (Figure 5B,C). After CAV1 knockdown, the proportion of clusters 4 and 9 significantly increased in MM1S cells, whereas cluster 1 decreased (Figure 5D; Figure S6D, Supporting Information). In RPMI 8226 cells, CAV1 knockdown increased the percentage of clusters 5 and 7 and reduced the proportion of clusters 8 and 15 (Figure 5E; Figure S6E, Supporting Information). Untargeted metabolomics analysis was also performed to further determine the impact of CAV1 inhibition on cell metabolism. Metabolite profiles were thoroughly compared after CAV1 knockdown. 124 and 98 metabolites were significantly up‐ or down‐regulated by CAV1 inhibition, respectively (Figure S7A, Supporting Information). The subclasses of these metabolites were summarized and the most important metabolites involved in CAV1 knockdown were displayed (Figure S7B–E, Supporting Information). KEGG enrichment analysis revealed that CAV1 mainly regulates metabolism, especially amino acid metabolism (Figure 5F,G). Moreover, using random forest analysis, the top ten metabolites that are mostly related to CAV1 inhibition in the positive (POS) or negative (NEG) ion model were identified (Figure 5H). These results indicate that targeting CAV1 also reprograms the metabolism, especially amino acid metabolism, in MM cells.

### 2.6 CAV1 Inhibition Regulates Glutamine Metabolism and Enhances Chemosensitivity to Bortezomib In Vivo

**Figure 6:**
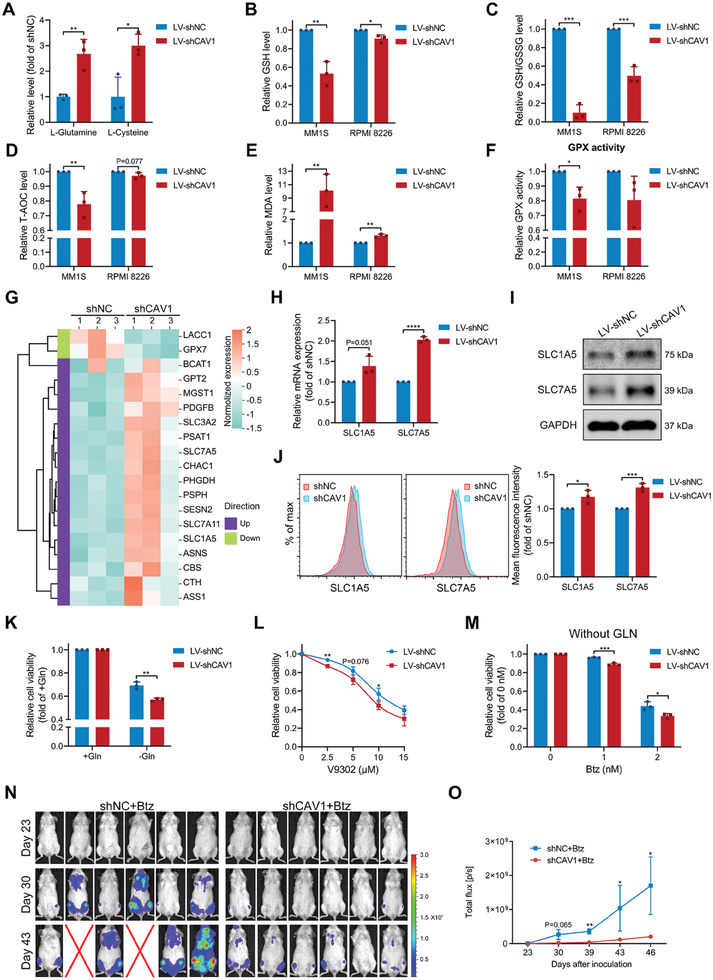
Targeting CAV1 regulated glutamine metabolism and enhanced the therapeutic efficiency of bortezomib. A) The relative level of L‐Glutamine and L‐Cysteine in MM1S cells expressing shNC or shCAV1. B) Relative GSH level in MM1S or RPMI 8226 cells expressing shNC or shCAV1. C) Relative GSH/GSSG ratio level in MM1S or RPMI 8226 cells expressing shNC or shCAV1. D and E) Relative total antioxidant capacity (T‐AOC) and malondialdehyde (MDA) levels in MM1S or RPMI 8226 cells expressing shNC or shCAV1. F) Relative GPX activity in MM1S or RPMI 8226 cells expressing shNC or shCAV1. G) A heatmap showing the expression of cell metabolism‐related genes in MM cells expressing shNC or shCAV1. H) mRNA expression of SLC1A5 and SLC7A5 in MM1S cells expressing shNC or shCAV1 was determined using qRT‐PCR. I) Protein expression of SLC1A5 and SLC7A5 in MM1S cells expressing shNC or shCAV1 was determined using a western blot. J) The total expression of SLC1A5 and SLC7A5 in MM1S cells was determined using flow cytometry. K) MM1S cells expressing shNC or shCAV1 were cultured with or without L‐Glutamine (Gln, 4 mM) for 24 h, and cell viability was measured. L) MM1S cells expressing shNC or shCAV1 were treated with V9302 at the indicated concentrations for 48 h and the cell viability was measured. M) In the absence of L‐Glutamine, MM1S cells expressing shNC or shCAV1 were treated with bortezomib at the indicated concentration for 48 h and the cell viability was measured. N and O) B‐NDG mice were intravenously inoculated with MM1S‐GFP‐Luc cells expressing shNC (n = 6) or shCAV1 (n = 6) and after 24 days, bortezomib (0.5 mg kg^−1^) was intraperitoneally injected three times a week. N) The distribution of MM cells in these mice was measured at the indicated days after inoculation using a living imaging system. O)The total flux in each mouse after inoculation for the indicated days was determined (Error bar, mean ± SEM.). *p*‐values were calculated with unpaired two‐sided Student *t*‐test or Mann–Whitney U test. Error bar, mean ± SD. ^*^, *p* < 0.05; ^**^, *p* < 0.01; ^***^, *p* < 0.001; ^****^, *p* < 0.0001.

According to the metabolomics data, we also found that L‐Glutamine and L‐Cysteine, two fundamental elements for glutathione (GSH) biosynthesis, were significantly increased by CAV1 knockdown in MM1S cells (**Figure** **6**A). RNA sequencing data also suggested a correlation between CAV1 and GSH metabolism (Figure S8A,B, Supporting Information). Since GSH is a key modulator of redox homeostasis, the oxidative‐ and antioxidant‐related indicators were examined. CAV1 knockdown reduced overall GSH levels and the GSH/Oxidized glutathione (GSSG) ratio in MM cells (Figure 6B,C). Additionally, CAV1 inhibition reduced total antioxidant capacity and glutathione peroxidase (GPX) activity, and increased the lipid peroxidation level (Figure 6D–F). To investigate the related mechanism, genes involved in GSH metabolism were summarized according to the sequencing data (Figure 6G). GSEA analysis demonstrated that CAV1 is associated with glutamine family amino acid metabolic process and transport (Figure S8C, Supporting Information). Thus, the expression of SLC1A5, SLC7A5, and SLC3A2, which are related to glutamine and amino acid transport and regulated by CAV1, was further analyzed. CAV1 knockdown significantly increased the mRNA and protein levels of SLC1A5 (ASCT2) and SLC7A5 (LAT1) but did not affect the overall level of SLC3A2 (Figure 6H–J; Figure S8D, Supporting Information). Since glutamine transport‐related proteins were modulated by CAV1, the response to glutamine in MM cells may also be changed. Indeed, MM cells with CAV1 deficiency were more sensitive to glutamine withdrawal and the glutamine transporter inhibitor V9302, but not to CB‐839, an inhibitor of glutaminase (Figure 6K,L; Figure S8E, Supporting Information). CAV1 inhibition in MM cells did not affect their sensitivity to bortezomib in the presence of glutamine, while it significantly increased the sensitivity to bortezomib in the absence of glutamine (Figure 6M; Figure S8F, Supporting Information). Importantly, CAV1 deficiency also suppressed MM cell growth treated with bortezomib in vivo, a condition without extra glutamine supplement (Figure 6N,O). These data suggest that although the redox homeostasis is disturbed by CAV1 inhibition, MM cells can increase glutamine transport by increasing SLC1A5 and SLC7A5 to maintain their survival in vitro.

### 2.7 6‐Mercaptopurine (6‐MP)‐ and Daidzin‐Mediated CAV1 Modulation Improves Bortezomib Treatment of MM

**Figure 7:**
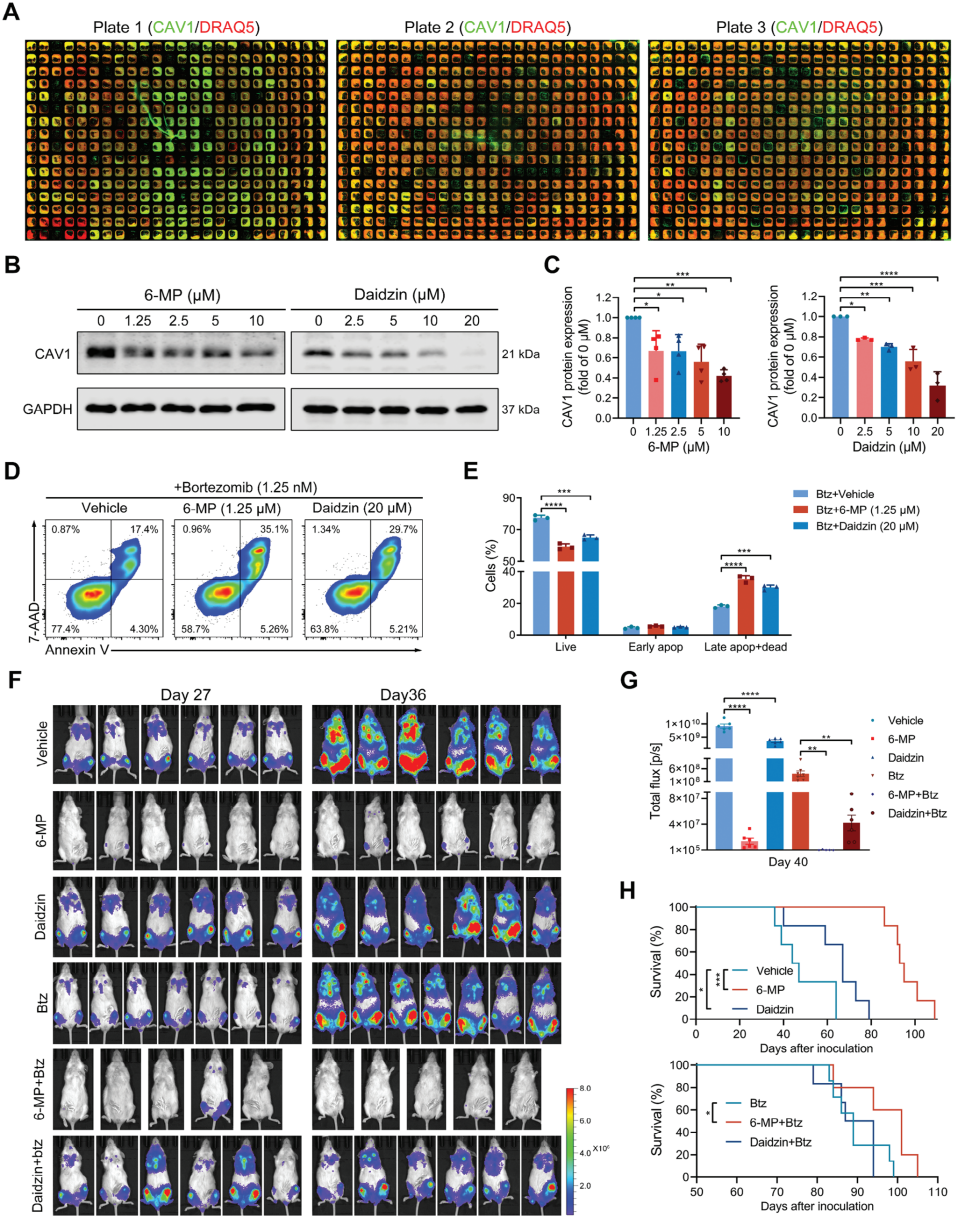
6‐MP and daidzin improve MM treatment with bortezomib. A) MM1S cells were treated with 1087 FDA‐approved drugs and the expression of CAV1 (green) was determined using in‐cell western. DRAQ5 (red) was used to stain DNA/total cells for normalization of cell numbers. B) MM1S cells were treated with 6‐MP or daidzin at the indicated concentrations for 48 h and the expression of CAV1 was detected using western blot. C) The signal density of CAV1 was quantified and normalized to GAPDH. D and E) MM1S cells were treated with bortezomib (1.25 nM) in combination with 6‐MP or daidzin for 48 h and cell apoptosis was determined using flow cytometry. Apop, apoptotic. F–H) B‐NDG mice were inoculated with MM1S‐Luc cells and randomly divided into six groups (n = 5–7). After 11 days of inoculation, these mice were intraperitoneally injected with 6‐MP (10–20 mg kg^−1^), daidzin (30 mg kg^−1^), or vehicle daily until day 67. After 15 days of inoculation, mice were intraperitoneally injected with bortezomib (0.5 mg kg^−1^) three times a week until day 67. F) The distribution of MM1S‐Luc cells in these mice was measured at the indicated days after inoculation using a living imaging system. G) The total flux in each mouse after inoculation for the 40 days was determined. Error bar, mean ± SEM. H) Kaplan–Meier curve showing mice survival. A logarithmic‐rank (Mantel–Cox) test was used to determine P values. *p*‐values were calculated with unpaired two‐sided Student *t*‐test or one‐way ANOVA test. Error bar, mean ± SD. ^*^, *p* < 0.05; ^**^, *p* < 0.01; ^***^, *p* < 0.001; ^****^, *p* < 0.0001.

Premised on our findings that CAV1 inhibition favors NK cell‐ and bortezomib‐based MM treatment, targeting CAV1 with pharmacological inhibitors may be an efficient strategy for MM treatment. Unfortunately, as of now, no specific inhibitors are available to target CAV1. Thus, a total of 1087 FDA‐approved drugs were screened using an in‐cell western assay to find those that inhibit CAV1 effectively to facilitate clinical translation (**Figure** **7**A). Most drugs promote the expression of CAV1, but one to two dozen drugs inhibit it (Figure S9A, Supporting Information). Among them, 6‐MP and daidzin efficiently inhibited CAV1 protein expression (Figure 7B,C; Figure S9B, Supporting Information) and enhanced bortezomib‐induced apoptosis in MM cells (Figure 7D,E; Figure S9C,D, Supporting Information). Both 6‐MP and daidzin inhibited MM growth and enhanced bortezomib‐induced inhibition of MM growth in vivo (Figure 7F,G; Figure S9E, Supporting Information). Additionally, these two drugs prolonged the survival of MM‐bearing mice when used alone. 6‐MP also further promoted the bortezomib‐mediated survival of MM‐bearing mice (Figure 7H). All these data suggest that 6‐MP and daidzin may serve as inhibitors of CAV1 and enhance MM treatment with bortezomib.

### 2.8 Statin‐Mediated CAV1 Modulation Enhances the Efficiency of Bortezomib in MM Treatment

**Figure 8:**
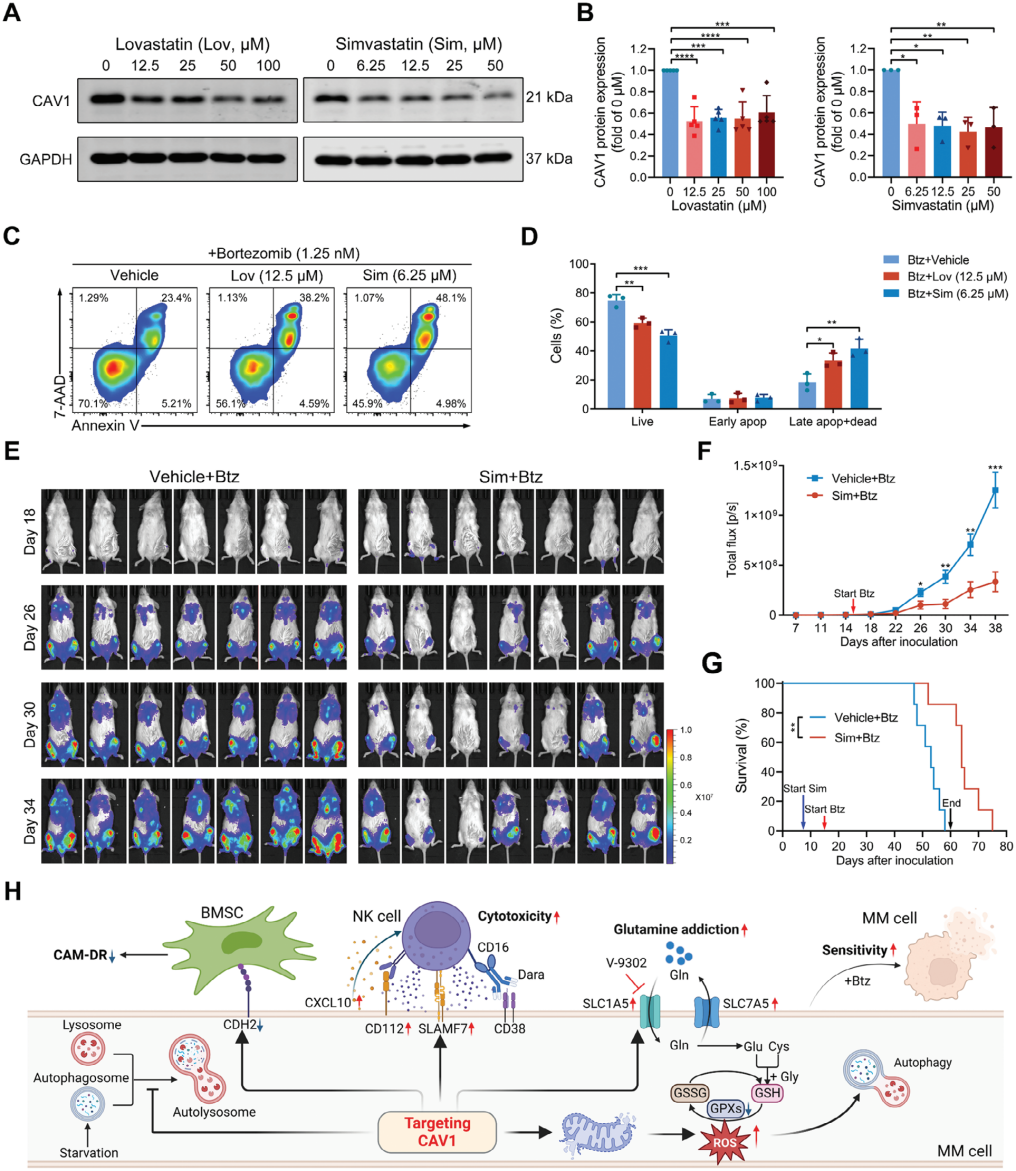
Statin‐mediated downregulation of CAV1 enhances the anti‐MM efficiency of bortezomib. A) MM1S cells were treated with lovastatin or simvastatin at the indicated concentration for 48 h and the expression of CAV1 was detected using western blot. B) The signal density of CAV1 was quantified and normalized to GAPDH. C,D) MM1S cells were treated with bortezomib (1.25 nM) in combination with lovastatin or simvastatin for 48 h and cell apoptosis was determined using flow cytometry. Apop, apoptotic. E–G) B‐NDG mice were inoculated with MM1S‐Luc cells and randomly divided into two groups. After 7 days of inoculation, these mice were intraperitoneally injected with simvastatin (20 mg kg^−1^, n = 7) or vehicle (n = 7) daily until day 60. After day 15 after inoculation, all these mice were intraperitoneally injected with bortezomib (0.5 mg kg^−1^) three times a week until day 60. (E) The distribution of MM1S‐Luc cells in these mice was measured at the indicated days after inoculation using a living imaging system. (F) The total flux in each mouse after inoculation for the indicated days was determined. Error bar, mean ± SEM. (G) Kaplan–Meier curve showing mice survival. A logarithmic‐rank (Mantel–Cox) test was used to determine P values. H) Schematic diagram showing how targeting CAV1 facilitates MM treatment through regulating cell adhesion, NK cell activation, autophagy, redox homeostasis, and glutamine transport. The figure was created with BioRender.com/v03k780. Gln, glutamine; Glu, glutamate, Cys, cysteine; Gly, glycine. *p*‐values were calculated with unpaired two‐sided Student *t*‐test or one‐way ANOVA test. Error bar, mean ± SD. ^*^, *p* < 0.05; ^**^, *p* < 0.01; ^***^, *p* < 0.001; ^****^, *p* < 0.0001.

Widely used and FDA‐approved cholesterol‐depleting drugs, lovastatin and simvastatin, have been proven to efficiently modulate CAV1 through the depletion of membrane cholesterol.^[^ ^6^, ^13^ ^]^ We sought to determine whether they would reduce CAV1 in MM cells and benefit MM treatment. Lovastatin and simvastatin significantly reduced the protein level of CAV1 in MM cells (**Figure** **8**A,B). Lovastatin alone failed to inhibit MM in vivo growth at a dose of 20 mg kg^−1^, a very high dose for in vivo experiments (Figure S10A,B, Supporting Information). However, lovastatin and simvastatin enhanced bortezomib‐induced apoptosis of MM cells (Figure 8C,D) and NK‐cell mediated killing of MM cells in vitro (Figure S10C,D, Supporting Information). Furthermore, simvastatin significantly enhanced the efficiency of bortezomib for MM treatment in vivo (Figure 8E–G). These results indicate that CAV1 inhibition by statins appears to offer a promising strategy to enhance the therapeutic effect of bortezomib for MM treatment.

## 3 Discussion

Benefiting from the development of novel therapeutic strategies, such as proteasome inhibition, immunomodulation, and monoclonal antibody therapy, survival in multiple myeloma has improved significantly in the last 15 years.^[^ ^14^ ^]^ However, the efficacy of these newly developed approaches for MM treatment is compromised by several mechanisms, including cell adhesion‐mediated drug resistance, downregulation of immunostimulatory markers, and reprogramming of metabolism.^[^ ^15^ ^]^ Identifying an effective target to reverse and enhance the therapeutic effects of these new therapies is highly desirable. In the work at hand, we demonstrated that targeting CAV1 reduces cell adhesion to BMSCs, increases immunostimulatory surface markers and cytokines, disturbs redox homeostasis, inhibits autophagy flux, and reprograms glutamine metabolism in MM cells, thereby enhancing NK cell‐mediated cytotoxicity and bortezomib sensitivity (Figure 8H), and thus serving as a promising novel therapeutic approach for MM treatment.

MM cells interact with components in the BM microenvironment to support their own survival and growth.^[^ ^16^ ^]^ Specifically, MM cells adhere to the extracellular matrix or BMSCs in the bone marrow through adhesion molecules, such as integrin family members,^[^ ^17^ ^]^ CD138,^[^ ^17a^ ^]^ CD44,^[^ ^17^, ^18^ ^]^ vascular cell adhesion molecule‐1,^[^ ^19^ ^]^ lymphocyte function‐associated antigen‐1,^[^ ^18^ ^]^ and intercellular adhesion molecule‐1,^[^ ^20^ ^]^ leading to the enhancement of their survival and CAM‐DR. In the present study, CAV1 inhibition significantly down‐regulated the expression of adhesion molecules, especially type‐I cadherins CDH2, CDH3, and CDH4. Among them, CDH2 (N‐cadherin) is associated with poor prognosis of MM patients and contributes to the adhesion of MM cells to BMSCs.^[^ ^9^ ^]^ Thus, CAV1 suppression likely reduces cell adhesion and drug resistance by decreasing surface N‐cadherin. CAV1 is generally considered a membrane protein that functions in membrane trafficking and signaling.^[^ ^21^ ^]^ As a result, it may influence signaling pathways that govern transcription factors or epigenetic modifiers, thereby regulating CDH2 mRNA transcriptions. Additionally, it may participate in the sorting and release of microRNAs that target CDH2 mRNAs via macrovesicles.^[^ ^22^ ^]^ However, further research is necessary to determine the detailed mechanisms by which CAV1 regulates CDH2.

The new therapeutic frontiers involve the use of new FDA‐approved MoAbs, such as daratumumab, isatuximab, and elotuzumab, which target CD38 or SLAMF7 for MM treatment.^[^ ^23^ ^]^ Even for advanced MM patients, MoAbs have shown good results in clinical therapy.^[^ ^24^ ^]^ MoAbs mediate MM cell death mainly through antibody‐dependent cell‐mediated cytotoxicity, antibody‐dependent cell phagocytosis, and complement‐dependent cytotoxicity.^[^ ^25^ ^]^ The therapeutic efficiency of MoAbs largely relies on the expression of their targets on the MM cell surface and the activation of effector immune cells, especially NK cells. SLAMF7 serves as a self‐ligand and has a unique co‐stimulatory function in NK cells.^[^ ^26^ ^]^ As a clinically approved therapy target, SLAMF7 on the MM cell surface directly binds to SLAMF7 on NK cell or its MoAbs followed by binds to FcgRIIIa/CD16 on NK cells, resulting in NK cell‐mediated MM cell killing.^[^ ^24c^ ^]^ Elevated surface SLAMF7 by CAV1 knockdown, even in RPMI8226 cells expressing low endogenous levels of SLAMF7, would promote the SLAMF7‐mediated interaction between NK and MM cells and thus enhance cytotoxicity against MM cells. CD112 is a ligand of CD226 which is highly expressed in NK and T cells.^[^ ^27^ ^]^ The binding of CD112–CD226 stimulates the NK cell‐mediated cytotoxicity.^[^ ^28^ ^]^ As a chemokine, CXCL10 has been shown to activate NK cells and enhance their ability to kill dormant tumor cells.^[^ ^29^ ^]^ Therefore, CAV1 inhibition‐enhanced anti‐MM activation of NK cells would result from the increase of SLAMF7, CD112, and CXCL10. Since CD38 is the target of daratumumab, the enhancement of daratumumab‐mediated ADCC may be attributed to the increased levels of CD38 caused by CAV1 inhibition.

In many types of cancer cells, the metabolic and mitochondrial functions are generally reprogrammed to ensure the production of vital molecules and to maintain mitotic signals for their proliferation.^[^ ^30^ ^]^ To maximize immunoglobulin production, plasma cells increase oxidative phosphorylation in mitochondria, resulting in the generation of ROS.^[^ ^31^ ^]^ ROS can contribute to normal cellular functions at mild levels, but excessive levels can cause cell stress and oxidative damage, ultimately leading to cell death.^[^ ^32^ ^]^ Upon activation, autophagy can remove ROS from the cells and function as an effective strategy to maintain redox homeostasis.^[^ ^33^ ^]^ In the present study, CAV1 inhibition increased ROS but did not induce cell death in vitro, possibly due to the modulation of autophagy and reprogramming of the GSH system, an important regulator of redox homeostasis.^[^ ^34^ ^]^ The GSH system consists of GSH, GPX, and glutathione‐transferases that reduce hydrogen peroxides or hydroperoxides using GSH as a substrate to inhibit ROS production. GSH synthesis requires cysteine, glycine, and glutamate, while glutamine is a vital precursor to glutamate.^[^ ^35^ ^]^ MM cells highly express glutaminase but not glutamine synthetase, making them particularly dependent on glutamine from the extracellular environment.^[^ ^36^ ^]^ It has already been demonstrated that glutamine depletion and inhibition of glutamine metabolism inhibit MM cell growth and enhance sensitivity to anti‐MM drugs.^[^ ^37^ ^]^ The glutamine transporters LAT1 (SLC7A5), SLC3A2, and ASCT2 (SLC1A5) efficiently transport glutamine across the cell membrane.^[^ ^38^ ^]^ In our study, CAV1 knockdown further enhanced glutamine addiction by increasing ASCT2 and LAT1 and thus supporting redox homeostasis and cellular survival in vitro. Despite surviving the disturbance of redox homeostasis, these CAV1‐deficient MM cells were more sensitive to autophagy induction, glutamine depletion, and glutamine transporter inhibition, which partly explains their reduced growth and increased sensitivity to bortezomib in vivo.

As an old anticancer drug, 6‐MP inhibits protein synthesis, DNA synthesis, and RNA synthesis, triggering apoptosis. This drug has been approved by the FDA for treating acute lymphoblastic leukemia, lymphoma, and other immune‐related diseases.^[^ ^39^ ^]^ Daidzin is a naturally occurring product from Kudzu roots that inhibits aldehyde dehydrogenase‐2, an enzyme crucial to alcohol metabolism.^[^ ^40^ ^]^ It is the 7‐O‐glucoside of daidzein which has cholesterol‐lowering effects.^[^ ^41^ ^]^ The reduction of CAV1 by daidzin may be caused by cholesterol distribution. These two “old” FDA‐approved drugs inhibit CAV1 levels efficiently and improve the MM treatment with bortezomib, thus supporting their use in clinical practice. Statins are FDA‐approved drugs prescribed to millions of people worldwide for the treatment of hypercholesterolemia and are used in preclinical studies to modulate CAV1 protein levels.^[^ ^6^, ^13^, ^42^ ^]^ Even though statins‐targeted CAV1 is not specific, these studies have demonstrated that statins are potent inhibitors of CAV1. According to our data, statins also efficiently inhibit CAV1 levels in MM cells. The enhancement of therapeutic efficacy by CAV1 inhibition and statins upholds the translational relevance of statins in MM treatment. A variety of retrospective findings have demonstrated that receipt of statins is associated with lower risk and reduced mortality of MM patients.^[^ ^43^ ^]^ Additionally, our findings and others showed that statins can overcome drug resistance against bortezomib in MM,^[^ ^44^ ^]^ further supporting the clinical use of statins in MM treatment.

In conclusion, CAV1 is comprehensively involved in the regulation of cell adhesion, NK cell stimulation, autophagy, ROS generation, redox homeostasis, and glutamine transport and metabolism in MM cells, and thus serves as a potent therapeutic target for enhancing chemotherapy and immunotherapy for MM. Although these important biological processes are modulated by CAV1, the underlying molecular mechanisms involved in all these effects need to be clarified in the future. From the perspective of CAV1 modulation, our study helps guide the clinical translation of 6‐MP, daidzin, and statins, which are readily available and well‐tolerated drugs, for improving MM treatment.

## 4 Experimental Section

### Cell Culture

Human MM cell lines, including RPMI 8226, MM1S, and U266 cells were purchased from the China Center for Type Culture Collection (Wuhan, China) and cultured in RPMI 1640 medium (Invitrogen, Carlsbad, CA, USA) supplemented with 10% fetal bovine serum (FBS, TransGen Biotech, Beijing, China), 2mM L‐glutamine (Biological Industries, Beit HaEmek, Israel), and 100 U mL^−1^ penicillin/streptomycin (Biological Industries). LP‐1 was purchased from Cellcook (Guangzhou China) and cultured in IMDM (Biological Industries) supplemented with 20% FBS (TransGen Biotech), 2mM L‐glutamine (Biological Industries), and 100 U mL^−1^ penicillin/streptomycin (Biological Industries). The human natural killer (NK) cell line NK‐92 was purchased from Procell (Wuhan, China) and cultured in improved MEM culture medium (without nucleoside) supplemented with 12.5% FBS (Procell, Wuhan, China), 12.5% horse serum (HS, Procell) and 200U mL^−1^ recombinant IL‐2 (PeproTech, Cranbury, NJ, USA). Bone marrow (BM) samples were collected from newly diagnosed MM patients after patients’ informed consent and the studies were conducted in accordance with the Declaration of Helsinki. All studies that involved human samples were approved by the Ethical Committee for Clinical Medicine Research of The Third Affiliated Hospital of Sun Yat‐Sen University ([2022]02‐379) and the Medical Ethic Committee of Guangzhou Medical University. BM mononuclear cells (BMMCs) were isolated from BM samples using Lymphoprep (Stemcell Technologies, Inc., Vancouver, BC, Canada). BMMCs were cultured in OriCell Human MSC Culture medium (Cyagen Biosciences, Suzhou, China). BMSCs that adhered to the bottom of the flask were continuously cultured with a Human MSC Culture medium (Nuwacell, Anhui, China) and used within 10 passages.

### Primary NK Cell Culture and Purification

BMMCs were cultured with an NK serum‐free culture kit (ExCell Bio, Taicang, China) according to the manufacturer's instructions. After amplification for seven days, these iBMMCs mainly contain NK cells. The purified NK cells were obtained through positive selection using anti‐human CD56‐biotin (Biolegend, San Diego, CA, USA) and streptavidin MicroBeads (Miltenyi Biotec, Bergisch Gladbach, Germany), and negative selection using biotinylated antibodies against human CD3 (Biolegend) and streptavidin MicroBeads using LS Columns (Miltenyi Biotec) and a MACS separator (Miltenyi Biotec). The purity of the NK (CD56^+^CD3^−^) cells was determined to be >95% using flow cytometry. Purified NK cells were then expanded using an NK serum‐free culture kit and used within 20 days after in vitro culture.

### Regents and Antibodies

Bortezomib, daratumumab, elotuzumab, lovastatin, simvastatin, bafilomycin A1, V9302, and CB‐839 were purchased from Selleck Chemicals (Houston, TX, USA). Antibodies against glyceraldehyde‐3‐phosphate dehydrogenase (GAPDH, 5174S, 1:1000), CAV1 (3267, 1:1000), p62 (8025, 1:1000), and LC3‐I/II (12741, 1:1000), SLC3A2 (47213, 1:1000) were purchased from Cell Signaling Technology (Danvers, MA, USA). Antibodies against SLC7A5 (ab305251, 1:1000) and SLC1A5 (608051, 1:1000) were obtained from Abcam (Cambridge, United Kingdom) and BioLegend, respectively. IRDye 680RD or 800CW goat anti‐mouse/rabbit IgG secondary antibodies (1:10000) were purchased from LI‐COR Biosciences (Lincoln, NE, USA). IgG1 control antibody (BE0297) was purchased from BioXcell (Lebanon, NH, USA). An FDA‐approved drug library Mini (containing rack HYCPK 39383, 39384, 39385, 39386, 39387, 39388, 39389, 39390, 39391, 39392, 39393, 39394, 39395, 39396, 39397, 39398, 39399, 39400), 6‐mercaptopurine (6‐MP), and daidzin were purchased from MedChemExpress (Princeton, NJ, USA).

### Lentivirus Transfections

Lentivirus expressing GFP and shRNAs against negative control (shNC) or CAV‐1 (shCAV1, targeting CACCTTCACTGTGACGAAATACTGGTT) were purchased from GenePharma (Shanghai, China). Lentivirus transfections were performed as described previously.^[^ ^5^ ^]^ RPMI 8226, MM1S, LP‐1, or U266 cells were transduced with lentivirus and after 48 h, cells were treated with 1 µg mL^−1^ puromycin (Beyotime Biotechnology, Shanghai, China) and amplified in the presence of puromycin for extra 14 days. These cells were used for assessing the knockdown efficiency and further experiments.

### Cell Viability Assay

MM cells (5 × 10^5^ cells mL^−1^) stably expressing shNC or shCAV1 were treated with or without bortezomib at the indicated concentration for 48 h and the viability was measured with the Cell Titer glo Luminescent Viability assay (Promega, Madison, WI, USA). In the absence or presence of bortezomib, MM cells stably expressing shNC or shCAV1 were cultured with or without 2 mM L‐glutamine for 24 or 48 h and the cell viability was identified by the Cell Titer glo Luminescent Viability assay. All viability assays were determined in triplicate and replicated in three independent experiments.

### Quantitative Real‐Time PCR (qRT‐PCR)

Total RNA was extracted from MM cells transduced with lentivirus using FastPure Cell/Tissue Total RNA Isolation Kit V2 (Vazyme, Nanjing, China) and reverse‐transcribed using HiScript III RT SuperMix for qPCR (Vazyme) following the manufacturer's instructions. The expression level of mRNA was quantified by qRT‐PCR using ChamQ universal SYBR qPCR Master Mix regent (Vazyme) and specific primers (Table S1, Supporting Information). Relative mRNA expression was normalized to GAPDH or β‐actin using the 2^−ΔΔCt^ method.

### Apoptosis Analysis

Apoptosis analysis was performed as previously described. In brief, MM cells treated with the indicated drugs under the indicated conditions were collected and stained with Annexin V‐FITC/APC (Biolegend) and 7‐Aminoactinomycin D (7‐AAD, Biolegend). The apoptotic cells were detected using the CytoFLEX (Beckman Coulter, Brea, CA, USA) flow cytometry and analyzed using FlowJo software (TreeStar, Ashland, OR, USA).

### Adhesion and Co‐Culture Assays

For adhesion to BMSCs and co‐culture assays, 1 × 10^4^ BMSCs were seeded in 96‐well plates overnight. After the complete attachment of BMSCs, 1 × 10^5^ MM cells were added and incubated for 6 h. Non‐adherent cells were gently removed by washing with RPMI 1640 medium and the overall cell viability was determined by the Cell Titer glo Luminescent Viability assay. For co‐culture assays, MM cells were added to BMSCs for 2 h and treated with bortezomib at the indicated concentrations for another 48 h. Finally, the cell viability was measured using the Cell Titer glo Luminescent Viability assay. The viability of BMSCs without co‐culture was also determined and used to exclude the effect of BMSC viability. All viability assays were determined in triplicate and replicated in three independent experiments.

### RNA Sequencing and Analysis

Total RNA was extracted from RPMI 8226 and MM1S cells transduced with lentivirus expressing shNC or shCAV1. The RNA sequencing and analysis were conducted by LC‐Bio Technologies (Hangzhou, China). The GO and KEGG analyses were performed using an online analyzer SangerBox.com. The GSEA was performed on omicstudio.cn. Heatmaps and volcano plots were generated on chiplot.online.

### Western Blot Analysis

The MM cell lysate was isolated using RIPA buffer (Beyotime) containing a proteinase inhibitor cocktail (Beyotime). The protein concentration of the cell lysate was quantified by the Pierce BCA Protein Assay Kit (Thermo). The cell lysate with loading buffer was loaded onto SDS‐PAGE gels and subjected to electrophoresis. The proteins in the gel were then transferred to immunoblot PVDF membranes and the membranes were blocked with QuickBlock Blocking Buffer (Beyotime). The membrane was incubated with primary antibodies and fluorescent dye‐labeled secondary antibodies. The protein bands on the membrane were observed and captured by an Odyssey CLx imaging system (LI‐COR Biosciences).

### Flow Cytometry

For cell surface protein detection, MM cells were collected and stained with anti‐human CD319‐PE/Cyanine7 (BioLegend, San Diego, CA, USA), anti‐human CD95‐APC/Cyanine7 (BioLegend), anti‐human CD112‐PerCP/Cyanine5.5 (BioLegend), or anti‐human CD325 (CDH2)‐APC (BioLegend). For measuring total protein using flow cytometry, MM cells were fixed and permeabilized using Cytofix/Cytoperm Fixation and Permeabilization Solution (BD Biosciences, Franklin Lakes, NJ, USA), and intracellularly stained with purified anti‐SLC1A5, anti‐SLC7A5, or anti‐CAV1 antibody followed by APC‐labeled goat anti‐rat IgG (BioLegend) or Alexa Fluor 647‐labeled goat anti‐rabbit IgG (Beyotime). The fluorescence intensity in these cells was determined using the CytoFLEX flow cytometry and analyzed using FlowJo software. CAV1 detection using flow cytometry.

### Cytokine Array

RPMI 8226 cells expressing shNC or shCAV1 were adjusted to be consistent according to their cell number and viability. After 24 h of culture, the condition medium was collected and used for cytokine array analysis. The Proteome Profiler Human XL Cytokine Array Kit (R&D Systems, Minnesota, USA) was used based on the manufacturer's instructions. The pixel intensity of each cytokine was quantified using an Odyssey CLx imaging system.

### ROS Measurement

MM cells treated with or without the indicated drugs for the indicated times were incubated with CellROX Deep Red dye or mitoSOX Red Mitochondrial Superoxide Indicators (Thermo Fisher Scientific, Waltham, MA, USA) at a final concentration of 5 µM in RPMI 1640 without FBS at 37 °C for 30 min. The dead cells were stained using SYTOX Blue dead cell stain (Thermo Fisher Scientific). The ROS level indicated by CellROX Deep Red dye or mitoSOX Red in living (SYTOX Blue negative) cells was determined using the CytoFLEX flow cytometry and analyzed using FlowJo software.

### Measurement of GSH and GSSG, GPX Activity, Lipid Peroxidation, Total Antioxidant Capacity (T‐AOC)

The levels of GSH and GSSG, GPX activity, lipid peroxidation, and T‐AOC in MM cells expressing shNC or shCAV1 were measured using the GSH and GSSG Assay Kit (Beyotime), Cellular Glutathione Peroxidase Assay Kit with NADPH (Beyotime), Lipid Peroxidation MDA Assay Kit (Beyotime), and Total Antioxidant Capacity Assay Kit with ABTS method (Beyotime), respectively, following the manufacturer's instruction.

### In‐Cell Western Assay

MM1S cells (3 × 10^4^ cells/well) were planted into poly‐D‐lysine‐coated 384‐well plates and cultured for 4 h. These cells were treated with 1087 FDA‐approved drugs (1 µM, 0.2 µM, or 0.3 µg mL^−1^) for 48 h and fixed with 4% paraformaldehyde. Fixed cells were permeabilized with phosphate‐buffered saline (PBS) supplemented with 0.1% Triton X‐100 and blocked with Odyssey Blocking buffer (LI‐COR Biosciences) for 1 h at room temperature. After washing, these cells were incubated with anti‐CAV1 rabbit antibody overnight at 4 °C and subsequently with diluted IRDye 800CW goat anti‐rabbit IgG secondary antibody and DRAQ5 (BioLegend) in Odyssey blocking buffer for 1 h at room temperature. The plates were scanned using the Odyssey CLx imaging system and the fluorescent signals in the 700‐ and 800 nm channels of each well were quantified using Image Studio software (LI‐COR Biosciences).

### Untargeted Metabolomics

1 × 10^7^ MM1S cells expressing shNC or shCAV1 were collected and washed with PBS three times. Metabolite extraction from these cell pellets. Metabolites were separated using a chromatographic column and detected using a mass spectrometer. Metabolite extraction and detection, as well as metabolomics analysis, were completed by iProteome (Shanghai, China).

### NK‐Mediated Cytotoxicity Assay

NK‐92, iBMMCs, or purified NK cells were co‐cultured with MM cells expressing shNC or shCAV1 at the indicated ratio, in the presence or absence of 10 µg mL^−1^ human IgG1 control antibody, daratumumab or elotuzumab, for the indicated times. The cells were stained with anti‐human CD45‐pacific blue, Annexin V‐APC, and 7‐AAD. The apoptosis of MM (CD45^−^) cells was determined using the CytoFLEX flow cytometry.

### Mass Cytometry

Mass cytometry and data analysis were performed as described previously.^[^ ^45^ ^]^ MM cells expressing shNC or shCAV1 were co‐cultured with iBMMCs for 6 h and incubated with Cell‐ID Cisplatin (Standard BioTools) for 5 min. All these cells were fixed with Fix I buffer (Standard BioTools) for 10 min at RT and barcoded separately using the Cell‐ID 20‐Plex Pd Barcoding Kit (Standard BioTools). Barcoded samples were pooled together, incubated with Human TruStain FcX Fc receptor blocking solution (BioLegend) to lower nonspecific binding, and then stained with anti‐human CD107a‐FITC antibody (BioLegend). After washing, these samples were stained with metal isotope‐tagged antibodies against surface markers and FITC (Table S2, Supporting Information). These cells were then permeabilized with Maxpar Perm‐S buffer (Standard BioTools) and stained with anti‐human Perforin‐APC antibody (BioLegend). After washing, these cells were stained with anti‐human granzyme B‐173Yb (Standard BioTools) and anti‐APC‐176Yb antibodies (Standard BioTools) in Maxpar Perm‐S buffer. For measuring the levels of intracellular proteins of MM cell lines, MM cells expressing shNC or shCAV1 were cultured in a complete medium for 48 h and incubated with IdU (50 µM) for another 30 min. Before cell collection, Cell‐ID Cisplatin (5 µM) was added for 5 min to stain the dead cells. These cells were immediately fixed with Fix I buffer (Standard BioTools) and barcoded. Barcoded cells were pooled together and stained with metal isotope‐tagged antibodies against surface proteins and then with antibodies against intracellular proteins (Table S3, Supporting Information) after permeabilization. After antibody staining, cells were resuspended in Fix & Perm Buffer (Standard BioTools) containing Cell‐ID Intercalator‐Ir (125 nM, Standard BioTools) overnight at 4 °C.

### Mass Cytometry Data Acquisition and Analyses

Stained samples were washed and resuspended in ultrapure water supplemented with 15% EQ Four Element Calibration Beads (Standard BioTools). All samples were acquired using a Helios mass cytometer (Standard BioTools). CyTOF software 6.7 (Standard BioTools) was used to normalize and debarcode the data. These data were uploaded to cytobank.cn for high‐dimensional analysis, such as viSNE and FlowSOM after excluding cell debris and doublets.

### In Vivo Study

Female six‐ to eight‐week‐old NOD‐Prkdc^scid^ IL2rg^tm1^/Bcgen (B‐NDG) mice were purchased from Biocytogen (Wakefield, MA, USA) and were housed and treated following conditions approved by the Institutional Animal Caring and Using Committee of Guangzhou Medical University (GY2023‐781). A total of 5 × 10^6^ MM1S cells expressing GFP and luciferase (MM1S‐GFP‐Luc) and shNC or shCAV1, or MM1S‐luc cells were subcutaneously or intravenously injected into B‐NDG mice. Bortezomib or/and 6‐MP, or daidzin, or simvastatin were administered by intraperitoneal injection at the indicated times. These mice were intraperitoneally injected with 150 mg kg^−1^ D‐luciferin (PerkinElmer, Waltham, MA, USA), and the bioluminescence signal of MM1S cells was measured using an IVIS Lumina III in vivo imaging system (PerkinElmer). Hindlimb paralysis was used as the endpoint criterion. Tumor size was calculated at the indicated time points using the following standard formula: Volume = (length × width^2^)/2.

### Public Data Analysis

The public data analyzed in this study were obtained from Gene Expression Omnibus (GEO) at GSE4581, GSE9782, GSE2113, and GSE5900, and analyzed on a web tool, GenomicScape (genomicscape.com).^[^ ^46^ ^]^

### Statistical Analysis

The Shapiro–Wilk test was used to determine whether the data was normally distributed. A parametric test (Student's *t*‐test) was used to determine the statistical significance between two groups if the data was normally distributed. Otherwise, a nonparametric test (Mann–Whitney U test) was used. For multiple group comparisons, one‐way ANOVA was performed. Tests were performed using GraphPad Prism software (Version 9, GraphPad Software, La Jolla, CA, USA). Error bars represent the mean ± standard deviation (SD) or standard error of the mean (SEM) as indicated. *p* < 0.05 was considered statistically significant.

## Conflict of Interest

The authors declare no conflict of interest.

## Author Contributions

D.Z., Z.D., and S.Z. contributed equally to this work. J.W. conceived the idea and supervised the overall project. D.Z., S.Z., J.H, H.Z., J.Z., and Z.L. implemented the experiments. Z.D. and J.W. analyzed the data and wrote the manuscript. E.M. provided critical suggestions and revised the manuscript. All authors read and approved the final manuscript.

## Supporting information



Supporting Information

## Data Availability

The data that support the findings of this study are available from the corresponding author upon reasonable request.
