# Supplementary material for: Targeting Caveolin‐1 in Multiple Myeloma Cells Enhances Chemotherapy and Natural Killer Cell‐Mediated Immunotherapy
Source: Adv Sci (Weinh). 2024 Dec 4;12(4):2408373. doi: 10.1002/advs.202408373 (PMC11789597; doi:10.1002/advs.202408373)
Supplement: Supplementary file 1 — Supporting Information [file ADVS-12-2408373-s001.pdf]

ADVANCED SCIENCE
Open Access

Supporting Information

for *Adv. Sci.*, DOI 10.1002/advs.202408373

Targeting Caveolin-1 in Multiple Myeloma Cells Enhances Chemotherapy and Natural Killer Cell-Mediated Immunotherapy

*Dewen Zhan, Zhimin Du, Shang Zhang, Juanru Huang, Jian Zhang, Hui Zhang, Zhongrui Liu, Eline Menu and Jinheng Wang**

Supporting Information

Targeting Caveolin-1 in Multiple Myeloma cells Enhances Chemotherapy and Natural Killer Cell-Mediated Immunotherapy

Dewen Zhan, Zhimin Du, Shang Zhang, Juanru Huang, Jian Zhang, Hui Zhang, Zhongrui Liu, Eline Menu, and *Jinheng Wang**

Supplementary Figures

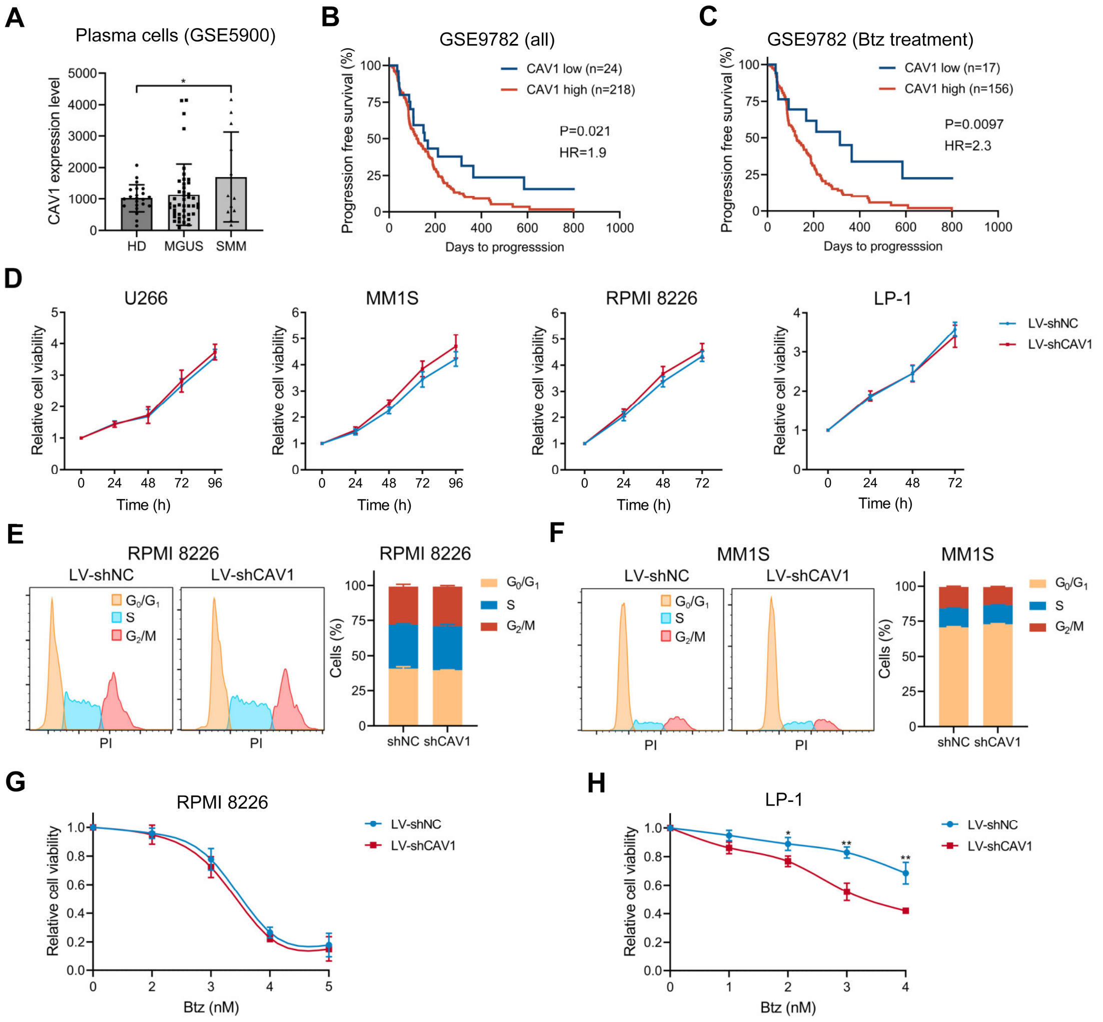

Figure S1. (A) CAV1 expression (GSE5900) in CD138$^+$ plasma from healthy donors (HD, n=22), MGUS (n=44), and smoldering MM (SMM, n=12). (B and C) Progression-free survival (PFS) according to high and low CAV1 expression in MM cells were

determined in all MM patients (GSE9782) or those treated with bortezomib (Btz) using Log-rank (Mantel-Cox) test (optimal cut-off points). (D) Growth curves of U266, MM1S, RPMI 8226, and MM1S cells expressing shRNAs against NC (shNC) or CAV1 (shCAV1) in vitro. (E and F) Cell cycle of (E) RPMI 8226 and (F) MM1S cells expressing shNC or shCAV1 were determined using flow cytometry. (G and H) RPMI 8226 and LP-1 cells expressing shNC or shCAV1 were treated with bortezomib at the indicated concentrations for 48 hours and the cell viability was examined. Error bar, mean ± SD. *, $P < 0.05$; **, $P < 0.01$.

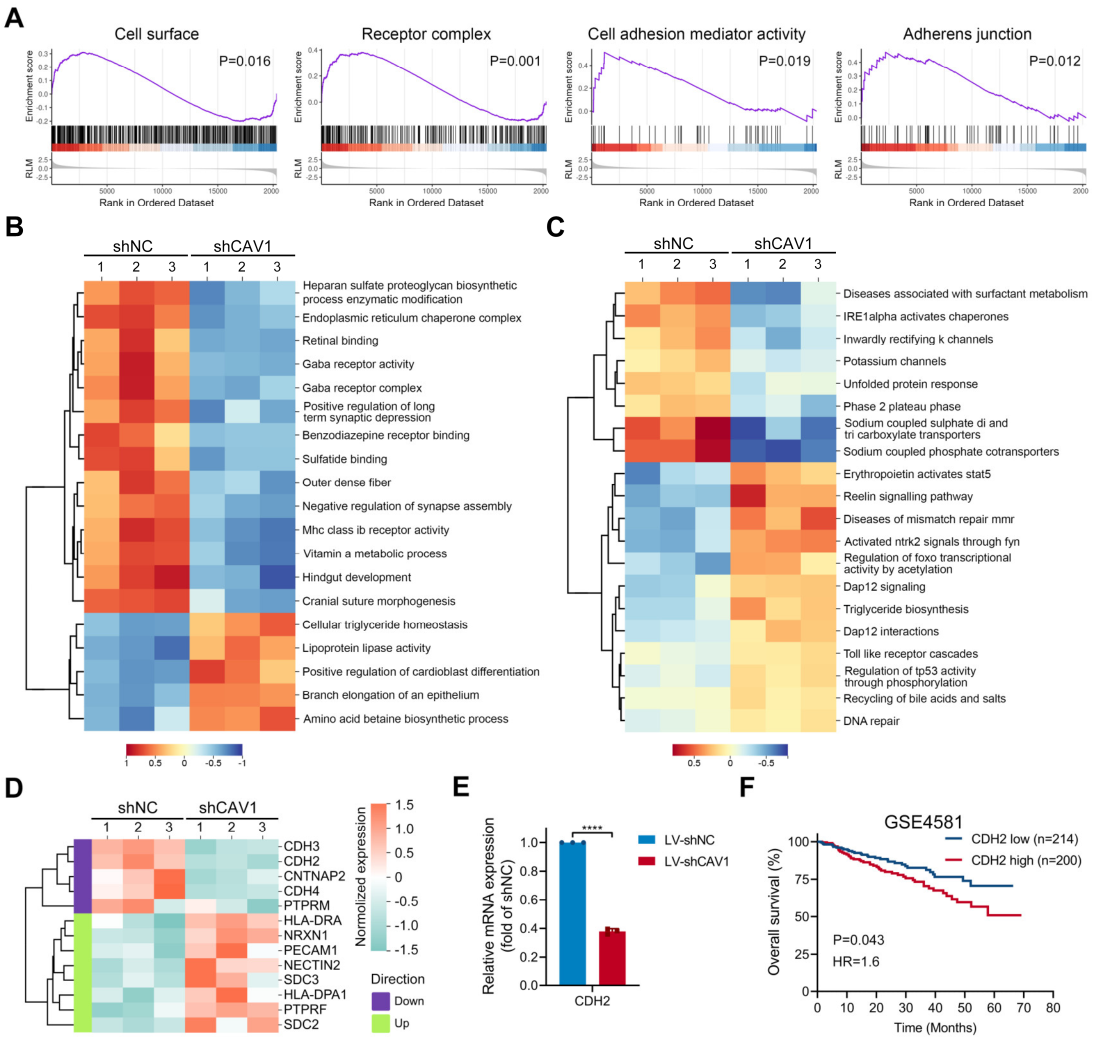

Figure S2. (A) GSEA analysis (GO terms) of genes regulated by CAV1 in RPMI 8226

cells. (B and C) Based on GSVA analysis, (B) GO terms and (C) Reactome terms significantly regulated by CAV1 in RPMI 8226 cells were showed by heatmaps. (D) A heatmap showing the expression of cell adhesion molecules in MM cells expressing shNC or shCAV1. (E) mRNA expression of CDH2 in MM cells expressing shNC or shCAV1 was determined using qRT-PCR. (F) Overall survival according to high and low CDH2 expression in plasma cells were determined in MM patients (GSE4581). Error bar, mean ± SD. ****, $P < 0.0001$.

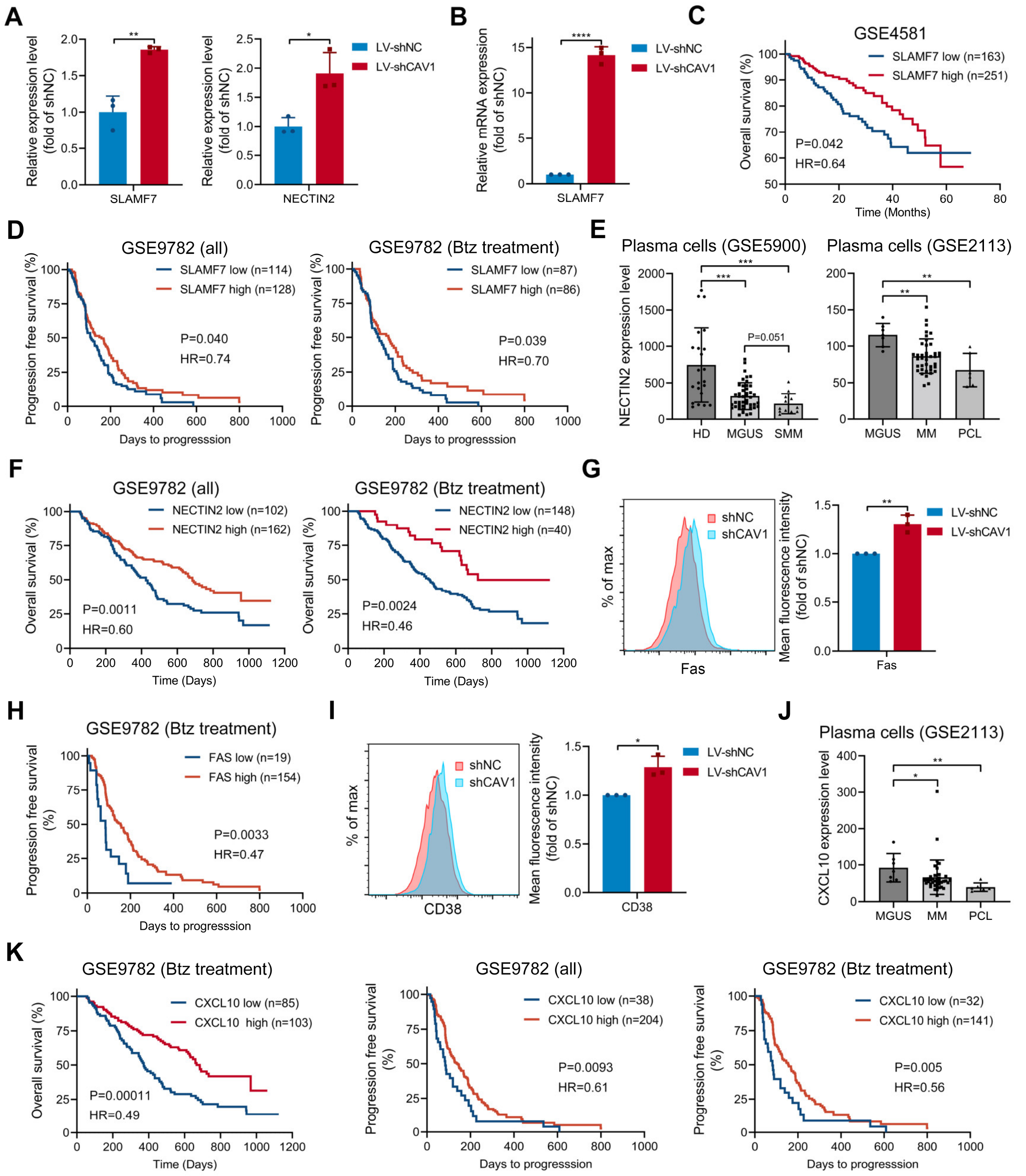

Figure S3. (A) Relative expression levels of SLAMF7 and NECTIN2 in MM cells expressing shNC and shCAV1 were examined using RNA sequencing. (B) mRNA expression of SLAMF7 in MM cells expressing shNC and shCAV1 were determined using qRT-PCR. (C) Overall survival (OS) according to high and low SLAMF7 expression in plasma cells were determined in MM patients (GSE4581). (D) PFS according to high and low SLAMF7 expression in plasma cells were determined in MM patients or those treated with Btz (GSE9782). (E) NECTIN2 expression (GSE5900,

203148_at) in $CD138^+$ plasma cells from HD (n=22), MGUS (n=44), SMM (n=12) patients and its expression (GSE2113, 203149_at) in $CD138^+$ plasma cells from MGUS (n=7), MM (n=39) and plasma cell leukemia (PCL, n=6) patients. (F) OS according to high and low NECTIN2 expression in plasma cells were determined in MM patients or those treated with Btz (GSE9782). (G) Surface level of Fas in MM cells expressing shNC or shCAV1 was determined using flow cytometry. (H) PFS according to high and low FAS expression in plasma cells were determined in MM patients treated with Btz (GSE9782). (I) Surface level of CD38 in MM cells expressing shNC or shCAV1 was determined using flow cytometry. (J) CXCL10 expression (GSE2113) in $CD138^+$ plasma cells from MGUS (n=7), MM (n=39) and PCL (n=6) patients. (K) OS and PFS according to high and low CXCL10 expression in plasma cells were determined in MM patients or those treated with Btz (GSE9782). Error bar, mean ± SD. *, $P < 0.05$; **, $P < 0.01$; ***, $P < 0.001$; ****, $P < 0.0001$.

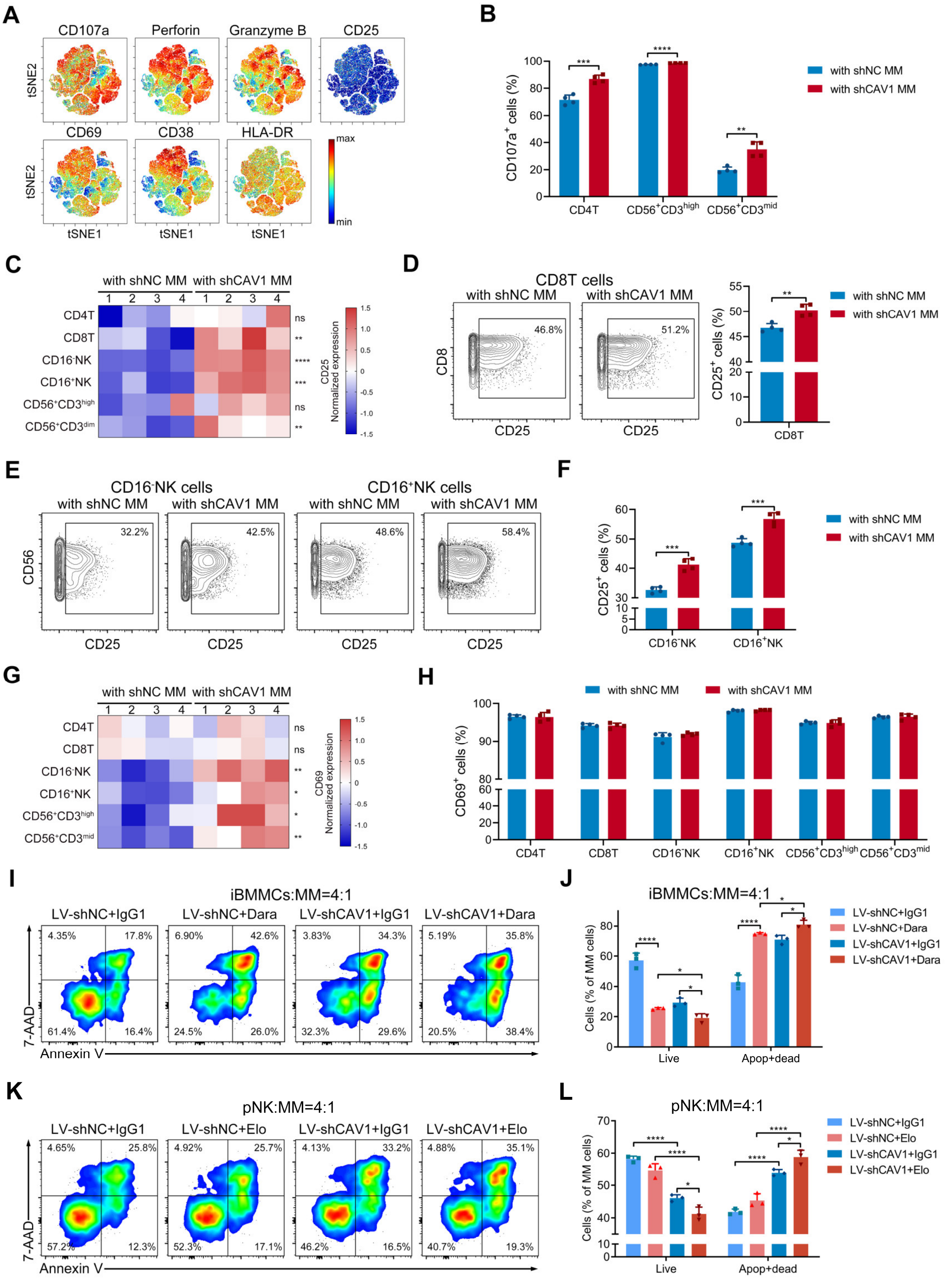

Figure S4. (A) viSNE maps colored by the normalized expression of the indicated markers in iBMMCs ($CD45^+$). (B) Bar plots showing the frequencies of the $CD107a^+$ cells in CD4 T, $CD56^+CD3^{high}$, and $CD56^+CD3^{mid}$ cell subsets. (C) A heatmaps showing the normalized mean expression of the CD25 in seven cell subsets. (D) Representative

contour plots for CD25 in CD8T cells after co-cultured with MM cells expressing shNC or shCAV1. (E) Representative contour plots for CD25 in $CD16^+$ or $CD16^-$ NK cells after co-cultured with MM cells expressing shNC or shCAV1. (F) Bar plots showing the frequencies of the $CD25^+$ cells in NK cell subsets. (G) A heatmaps showing the normalized mean expression of the CD69 in seven cell subsets. (H) Bar plots showing the frequencies of the $CD69^+$ cells in CD4 T, CD8 T, NK, $CD56^+CD3^{high}$, and $CD56^+CD3^{mid}$ cell subsets. (I and J) In presence of 10 μg/mL daratumumab (Dara) or isotype IgG1, iBMMCs ($CD45^+$) were co-culture with RPMI 8226 ($CD45^-$) cells expressing shNC or shCAV1 at the ratio of 4:1 for six hours and the apoptosis of MM cells were determined using flow cytometry. (K and L) In presence of 10 μg/mL elotuzumab (Elo) or isotype IgG1, pNK cells were co-culture with RPMI 8226 cells expressing shNC and shCAV1 at the ratio of 4:1 for six hours and the apoptosis of MM cells were determined using flow cytometry. Error bar, mean ± SD. *, $P < 0.05$; **, $P < 0.01$; ***, $P < 0.001$; ****, $P < 0.0001$.

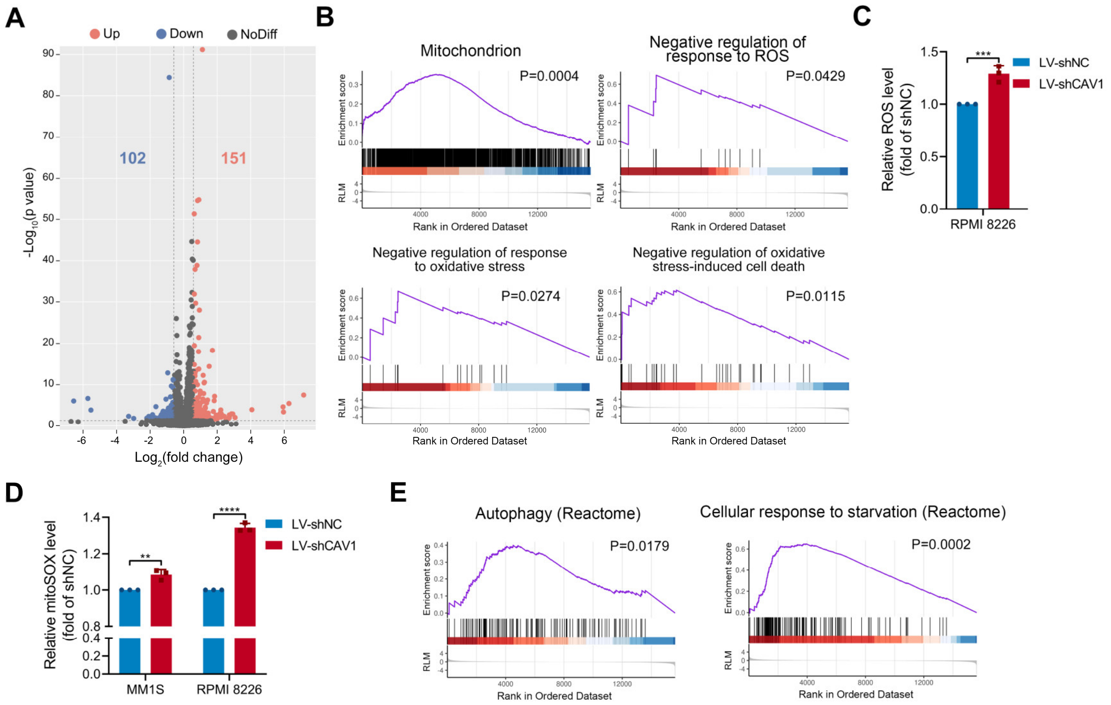

Figure S5. (A) Volcano plot showing the differential mRNA expression between MM1S expressing shNC and shCAV1. Red dots represent up-regulated genes and blue dots represent down-regulated genes. (B) GSEA analysis (GO terms) of genes regulated by CAV1 in MM1S cells. (C) RPMI 8226 cells expressing shNC or shCAV1 were incubated with CellROX Deep Red for 15 minutes and the ROS level was determined using flow cytometry. (D) MM1S or RPMI 8226 cells expressing shNC or shCAV1 were incubated with mitoSOX Red for 15 minutes and the mitochondria ROS level was determined using flow cytometry. (E) GSEA analysis (Reactome terms) of genes regulated by CAV1 in MM1S cells. Error bar, mean ± SD. **, $P < 0.01$.

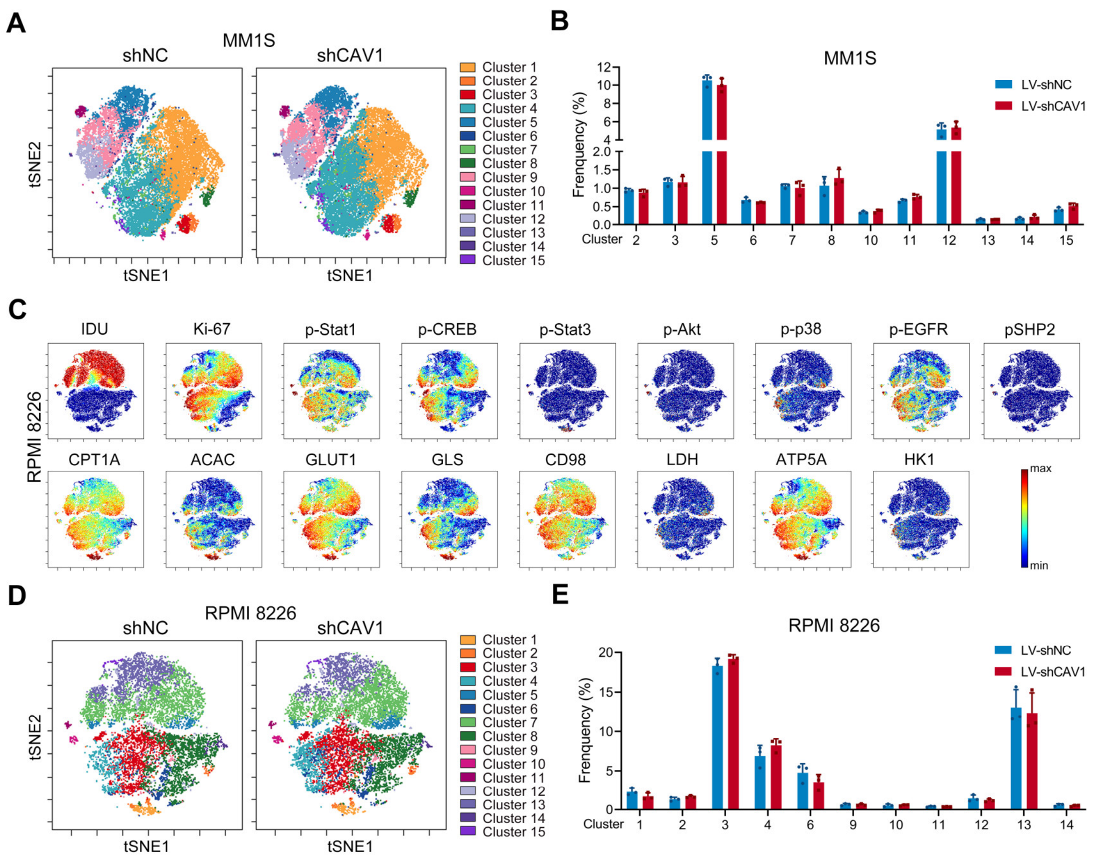

Figure S6. (A-E) Multiple cell metabolism-related protein in MM1S or RPMI 8226 cells expressing shNC or shCAV1 were analyzed using mass cytometry. (A) viSNE map colored by 15 populations of MM1S cells after FlowSOM clustering. (B) Bar plots showing the frequencies of the indicated cell clusters in MM1S expressing shNC and shCAV1. (C) viSNE maps colored by the normalized expression of the indicated markers in RPMI 8226 cells. (D) viSNE map colored by 15 populations of RPMI 8226 cells after FlowSOM clustering. (E) Bar plots showing the frequencies of the indicated cell clusters in RPMI 8226 expressing shNC and shCAV1. Error bar, mean ± SD.

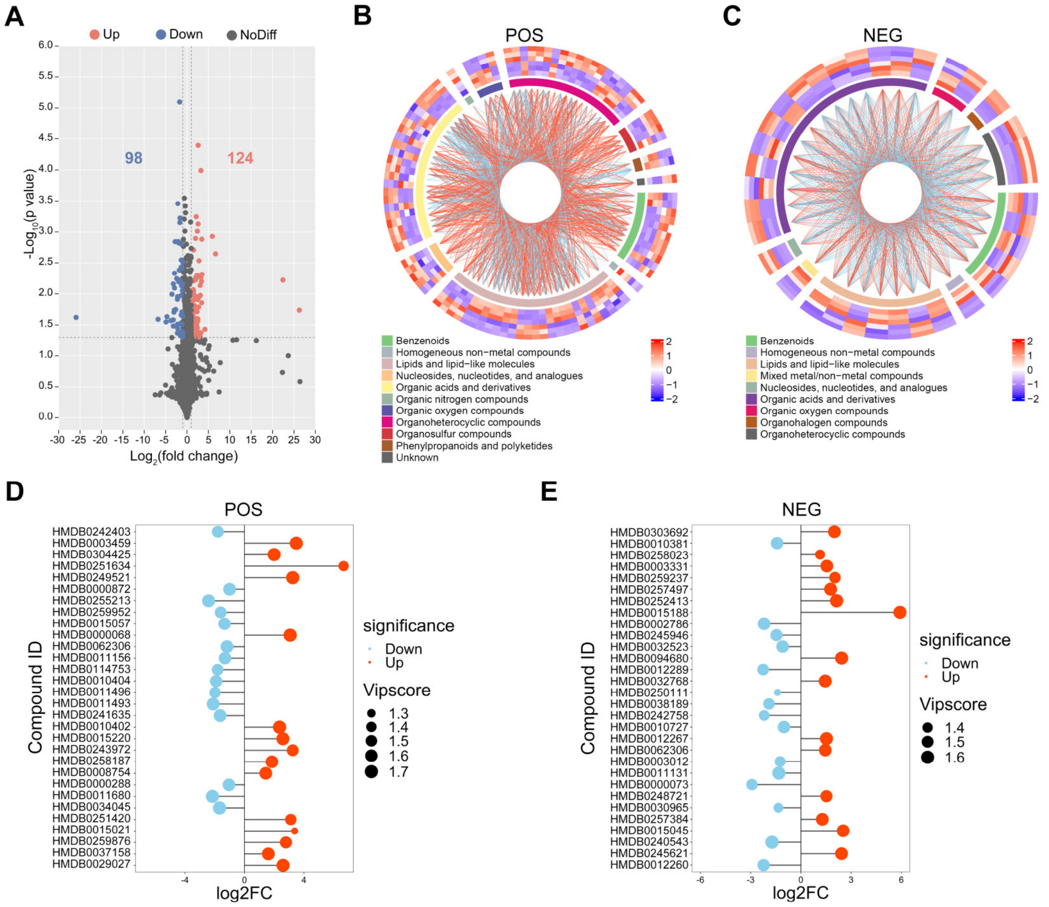

Figure S7. (A) Volcano plot showing the differential metabolites between MM1S expressing shNC and shCAV1. Red dots represent up-regulated metabolites and blue dots represent down-regulated metabolites. (B and C) Circle heatmap showing the normalized metabolites levels and their subclass in RPMI 8226 expressing shNC and shCAV1 under (B) POS and (C) NEG model. (D and E) Lollipop Chart showing the top 15 significant changed and the most important metabolites in MM1S cells expressing shNC and shCAV under (D) POS and (E) NEG model.

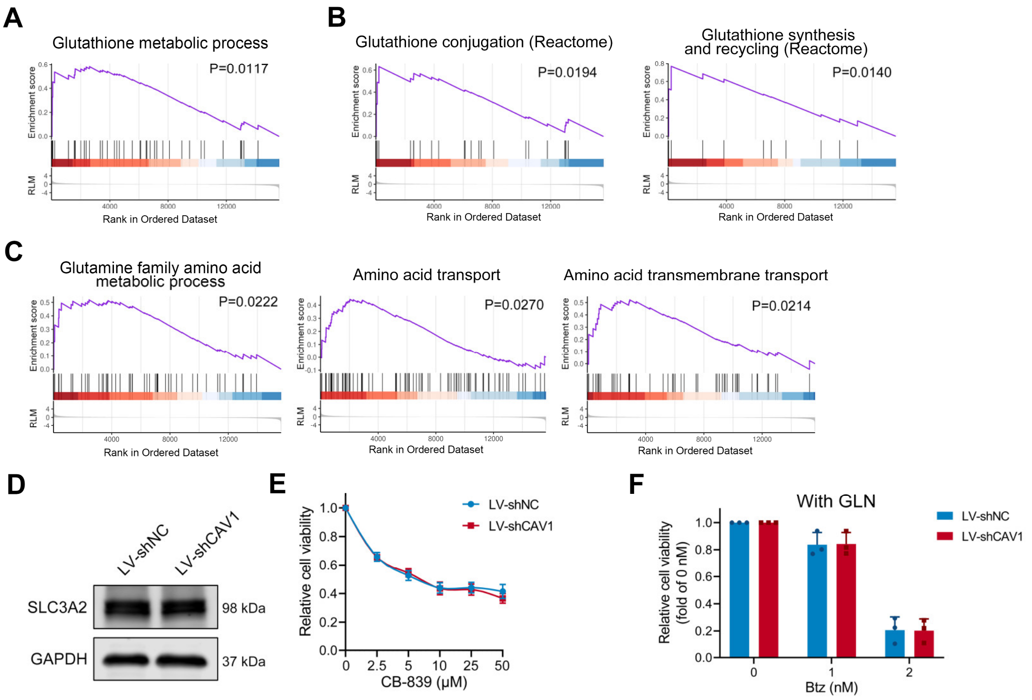

Figure S8. (A and C) GSEA analysis (GO terms) of genes regulated by CAV1 in MM1S cells. (B) GSEA analysis of genes regulated by CAV1 (Reactome database) in glutathione conjugation, glutathione synthesis and recycling. (D) Protein expression of SLC3A2 (CD98) in MM cells expressing shNC or shCAV1 was detected using western blot. GAPDH was included as a loading control. (E) MM1S cells expressing shNC or shCAV1 treated with CB-839 at the indicated concentration for 48 hours and the cell viability was measured. (F) In the presence of L-glutamine, MM1S cells expressing shNC or shCAV1 were treated with bortezomib at the indicated concentration for 48 hours and the cell viability was measured. Error bar, mean ± SD.

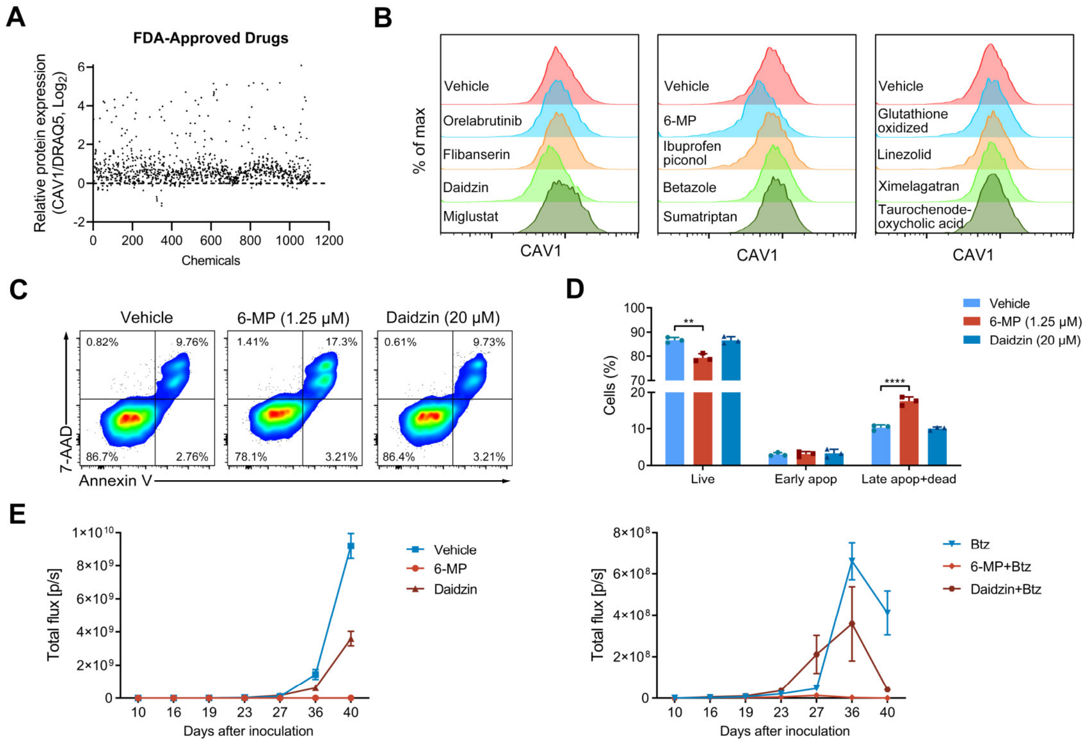

Figure S9. (A) 1087 FDA-approved drugs were subjected to screen inhibitors of CAV1 using in-cell western. The expression of CAV1 was normalized to DRAQ5. (B) MM1S cells were treated with the indicated drugs for 48 hours and the level of CAV1 in these cells were determined using flow cytometry. (C and D) MM1S cells were treated 6-mercaptopurine (6-MP) or daidzin for 48 hours and cell apoptosis was determined using flow cytometry. Error bar, mean ± SD. (E) B-NDG mice were inoculated with MM1S-Luc cells and randomly divided into six groups (n=5-7). After 11 days of inoculation, these mice were intraperitoneally injected with 6-MP (10-20 mg/kg), daidzin (30 mg/kg), or vehicle daily until day 67. After 15 days of inoculation, mice were intraperitoneally injected with bortezomib (0.5 mg/kg) three times a week until day 67. Multiple myeloma burden in these mice was measured at the indicated days using a live imaging system. The total flux in each mouse after inoculation for the indicated days was determined. Error bar, mean ± SEM. **, $P < 0.01$; ****, $P < 0.0001$.

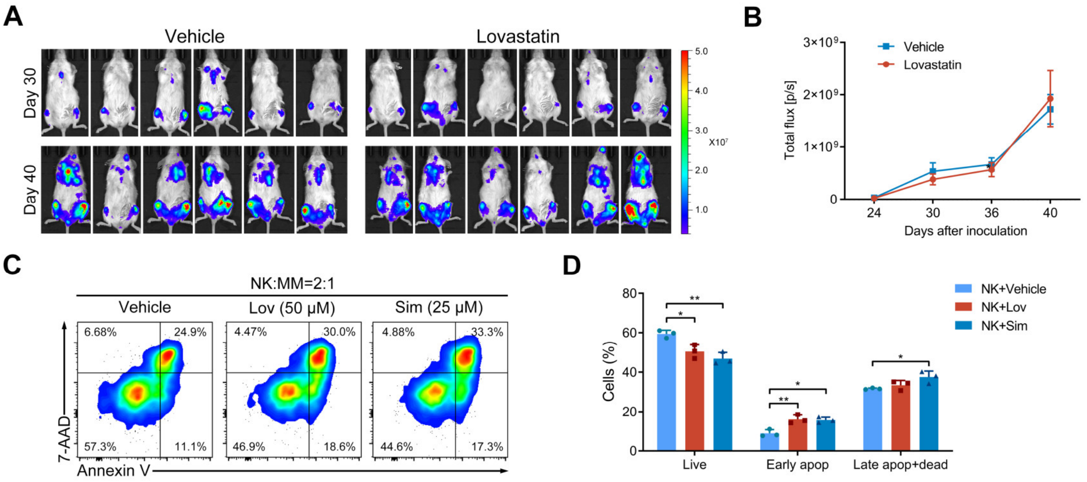

Figure S10. (A and B) B-NDG mice were inoculated with MM1S-Luc cells and randomly divided into two groups. After 25 days of inoculation, these mice were orally administrated with lovastatin (20 mg/kg, n=6) or vehicle (n=6) daily. (A) The distribution of MM1S-Luc cells in these mice was measured at the indicated days after inoculation using a living imaging system. (B) The total flux in each mouse after inoculation for the indicated days was determined. Error bar, mean ± SEM. (C and D) RPMI 8226 cells were treated with vehicle, lovastatin (lov), or simvastatin (sim) for 24 hours and co-cultured with NK92 cells for another 12 hours. The cell apoptosis was determined using flow cytometry. Apop, apoptotic. Error bar, mean ± SD. *, $P < 0.05$; **, $P < 0.01$.

Supplementary Tables

Table S1. List of primers for qRT-PCR.

Gene	Forward primer	Reverse primer
SLAMF7	CTGGAGAGAACACAGAGTACGACAC	CTGGCATCGTGAGCAGTGAGT
CAV1	AGCAGCCTCCCTGAAGACCA	CGGATAAAGATGTTGCCAGATGA
GAPDH	GAACGGGAAGCTCACTGG	GCCTGCTTCACCACCTTCT
ACTB	TCAAGATCATTGCTCCTCCTGAG	ACATCTGCTGGAAGGTGGACA
SLC1A5	ACTCGACAGGATATTGAGGGGA	TGCTGACACCAGGTTGGAAG
SLC7A5	CGTGGACTTCGGGAACTATCA	TGAACAGGGACCCATTGACG
CDH2	AACGCCAGGCCAAACAACT	TTCGTCGGATTCCCACAGG

Table S2. Mass cytometry antibody panel

Label	Target	Ab clone	Source
155Gd	CD56	B159	Standard BioTools
149Sm	CD25	2A3	Standard BioTools
154Sm	CD3	UCHT1	Standard BioTools
89Y	CD45	HI30	Standard BioTools
167Er	CD38	HIT2	Standard BioTools
151Eu	HLA-DR	G46-6	Standard BioTools
168Er	CD8a	SK1	Standard BioTools
174Yb	CD4	SK3	Standard BioTools
148Nd	CD16	3G8	Standard BioTools
160Gd	FITC	FIT-22	Standard BioTools
162Dy	CD69	FN50	Standard BioTools

Table S3. Mass cytometry antibody panel

Label	Target	Ab clone	Source
144Nd	CD98*	MEM-108	BioLegend
145Nd	CD138	DL-101	Standard BioTools
167Er	CD38	HIT2	Standard BioTools
149Sm	CD56 (NCAM)	NCAM16.2	Standard BioTools
Intracellular proteins			
141Pr	pSHP2 [Y580]	D66F10	Standard BioTools
146Nd	pEGFR [Y1068]	D7A5	Standard BioTools
152Sm	pAkt [S473]	D9E	Standard BioTools
153Eu	pStat1 [Y701]	58D6	Standard BioTools
156Gd	p-p38 [T180/Y182]	D3F9	Standard BioTools

158Gd	pStat3 [Y705]	4/P-Stat3	Standard BioTools
161Dy	Ki-67	B56	Standard BioTools
165Ho	pCREB [S133]	87G3	Standard BioTools
150Nd	CPT1A*	8F6AE9	Abcam
163Dy	HK1*	EPR10134(B)	Abcam
164Dy	ATP5A*	7H10BD4F9	Abcam
166Er	ACAC*	143	Invitrogen
171Yb	GLUT1*	EPR3915	Abcam
173Yb	GLS*	6H5L15	Invitrogen
175Lu	LDH*	98A-1F9BB1	Abcam

*These Abs were labeled using Maxpar Antibody Labeling Kit (Standard BioTools).
